# Functional Characterization of the Incomplete Phosphotransferase System (PTS) of the Intracellular Pathogen *Brucella melitensis*

**DOI:** 10.1371/journal.pone.0012679

**Published:** 2010-09-10

**Authors:** Marie Dozot, Sandrine Poncet, Cécile Nicolas, Richard Copin, Houda Bouraoui, Alain Mazé, Josef Deutscher, Xavier De Bolle, Jean-Jacques Letesson

**Affiliations:** 1 Research Unit in Molecular Biology (URBM), University of Namur, Namur, Belgium; 2 Laboratoire de Microbiologie et Génétique Moléculaire (INRA-CNRS-AgroParisTech), Thiverval-Grignon, France; Universidad Nacional, Costa Rica

## Abstract

**Background:**

In many bacteria, the phosphotransferase system (PTS) is a key player in the regulation of the assimilation of alternative carbon sources notably through catabolic repression. The intracellular pathogens *Brucella* spp. possess four PTS proteins (EI^Ntr^, NPr, EIIA^Ntr^ and an EIIA of the mannose family) but no PTS permease suggesting that this PTS might serve only regulatory functions.

**Methodology/Principal Findings:**

*In vitro* biochemical analyses and *in vivo* detection of two forms of EIIA^Ntr^ (phosphorylated or not) established that the four PTS proteins of *Brucella melitensis* form a functional phosphorelay. Moreover, *in vitro* the protein kinase HprK/P phosphorylates NPr on a conserved serine residue, providing an additional level of regulation to the *B. melitensis* PTS. This kinase activity was inhibited by inorganic phosphate and stimulated by fructose-1,6 bisphosphate. The genes encoding HprK/P, an EIIA^Man^-like protein and NPr are clustered in a locus conserved among α-proteobacteria and also contain the genes for the crucial two-component system BvrR-BvrS. RT-PCR revealed a transcriptional link between these genes suggesting an interaction between PTS and BvrR-BvrS. Mutations leading to the inactivation of EI^Ntr^ or NPr significantly lowered the synthesis of VirB proteins, which form a type IV secretion system. These two mutants also exhibit a small colony phenotype on solid media. Finally, interaction partners of PTS proteins were identified using a yeast two hybrid screen against the whole *B. melitensis* ORFeome. Both NPr and HprK/P were shown to interact with an inorganic pyrophosphatase and the EIIA^Man^-like protein with the E1 component (SucA) of 2-oxoglutarate dehydrogenase.

**Conclusions/Significance:**

The *B. melitensis* can transfer the phosphoryl group from PEP to the EIIAs and a link between the PTS and the virulence of this organism could be established. Based on the protein interaction data a preliminary model is proposed in which this regulatory PTS coordinates also C and N metabolism.

## Introduction

In order to successfully colonize an ecological niche, bacteria have to integrate different signals indicating environmental changes, and subsequently trigger an adequate adaptative response by modulating their cellular activities. The appropriate response to changes in nutrient availability, for example, relies on diversified mechanisms, including global regulation systems such as the phosphoenolpyruvate (PEP): carbohydrate phosphotransferase system (PTS). The PTS catalyzes the uptake and concomitant phosphorylation of carbohydrates and is composed of several proteins forming a phosphorelay transferring the phosphoryl group from PEP to the incoming sugar: (i) the general PTS proteins enzyme I (EI) and HPr are cytoplasmic components usually common to all PTS carbohydrates; (ii) the enzyme II complex is specific for one or several sugars and is generally composed of at least three domains (or distinct proteins) including the cytoplasmic EIIA and EIIB, and the membrane-crossing EIIC (sometimes also EIID) that constitutes the permease of the system [1], [2]. PTS proteins are usually phosphorylated on a conserved histidine, with the exception of most EIIB components that are phosphorylated on a cysteine. Besides its function in the transport and phosphorylation of carbon sources, the PTS plays a key role in the regulation of many aspects of bacterial physiology, including carbon catabolite repression (CCR) (for reviews see [1], [2], [3]).

Interestingly, a paralog of the classical PTS was proposed to function as a regulatory link between carbon and nitrogen metabolism. This system was first identified in *Escherichia coli* and called the nitrogen PTS (PTS^Ntr^) [4], [5], [6], [7]. The phosphoryl transfer chain of this system is composed of three proteins, EI^Ntr^ (encoded by *ptsP*), NPr (encoded by *ptsO*) and EIIA^Ntr^ (encoded by *ptsN*) that are the respective paralogs of EI, HPr, and EIIA of the fructose PTS family; however, they are not associated with PTS permeases [4], [5], [6], [7] but carry out multiple regulatory functions [8]. For example, the PTS^Ntr^ is involved in the regulation of genes related to nitrogen metabolism [9], [10], [11], [12]. Moreover, compared to EI, the EI^Ntr^ possesses an N-terminal extension homologous to the GAF N-terminal sensory domain of NifA from *Azotobacter vinelandii*, an activator that enhances transcription by σ^54^-associated RNA polymerase [13]. Finally, PTS^Ntr^ might favor the utilization of organic nitrogen compounds when bacteria are exposed to multiple carbon sources [4], [11], [12], [14] and is involved in maintaining K^+^ homeostasis in *E. coli* [15].

*Brucella* spp. are Gram negative intracellular pathogens belonging to the α-proteobacteria group which includes other bacteria interacting with eukaryotic hosts, such as *Agrobacterium tumefaciens* or *Sinorhizobium meliloti* [16]. They are responsible for brucellosis, a worldwide zoonosis that affects a broad range of mammals [17], and can also infect humans where it may cause Malta fever, a serious debilitating chronic disease [18]. Large-scale screens aiming at the isolation of attenuated transpositional mutants of *Brucella* spp. led to the identification of many genes involved in carbon and nitrogen metabolism [19], [20]. Moreover, genes encoding homologues of the three components of the *E. coli* PTS^Ntr^ were also isolated during these screens [19], [20], [21]. These data suggest that carbon and nitrogen metabolism might affect the virulence of *Brucella*.

The availability of the genome sequence of several *Brucella* species [22], [23], [24] allowed the identification of an additional PTS-related gene putatively encoding an EIIA belonging to the mannose PTS family. Moreover, a gene encoding a truncated homologue of HPr kinase/phosphorylase (HprK/P) was found in *Brucella* genomes [25], [26], [27]. In most firmicutes (Gram positive bacteria with low GC content), HprK/P catalyses the phosphorylation and dephosphorylation of a conserved serine residue in HPr (usually Ser46) [28], [29], [30]. In these bacteria, HPr phosphorylated on this conserved serine (P-Ser-HPr) is a central regulator of carbon metabolism mediating among others inducer exclusion and acting as a co-repressor of the catabolite control protein A (CcpA) during CCR [1], [31].

Similarly to *Brucella* spp, other α-proteobacteria including *S. meliloti* [32] and *A. tumefaciens* (S. Poncet, A. Khemiri and J. Deutscher, unpublished) possess the predicted PTS^Ntr^ proteins, as well as an EIIA^Man^-like protein and HprK/P, and lack PTS permeases. It was therefore suggested [25], [26], [27] that *Brucella* PTS proteins might form a phosphoryl transfer chain exclusively dedicated to regulatory functions. Interestingly, in these three bacteria, the genes *ptsO*, *ptsM* and *hprK* (encoding respectively NPr, an EIIA^Man^-like protein and HprK/P) are localized close to genes encoding (i) a two-component system involved in virulence or symbiosis (BvrR-BvrS in *Brucella* spp., ChvI-ChvG in *A. tumefaciens* and ChvI-ExoS- in *S. meliloti*) [33], [34], [35], (ii) S-adenosyl homocysteine hydrolase (SahH), an enzyme involved in the metabolism of methionine [36], [37] and (iii) PEP carboxykinase, a key enzyme of gluconeogenesis [38], [39], [40]. As previously proposed by Hu and Saier [27], the conservation of this genomic locus in several α-proteobacteria suggests a functional link between PTS, HprK/P and the neighboring genes in regulating carbon/nitrogen metabolism in these organisms.

In this report, we demonstrate by *in vitro* and *in vivo* experiments that the PTS proteins found in *B. melitensis* function in a phosphorelay. Moreover, we observed that NPr is phosphorylated not only by EI^Ntr^ on His-30, but also by HprK/P on a conserved serine (Ser-61). This latter phosphorylation slows the *in vivo* phosphotransfer to His66 in EIIA^Ntr^. We also demonstrated a transcriptional link between the PTS genes *ptsO*, *ptsM* and *hprK* and the two component system genes *bvrR/S* establishing a link between virulence and metabolism that was reinforced by the observation that both, *ptsP* and *ptsO* mutants, almost completely lost the synthesis of a type IV secretion system (T4SS). Finally by carrying out a yeast two hybrid screen against the whole *B. melitensis* ORFeome we identified several interaction partners of PTS proteins allowing us to propose a preliminary model of regulation for carbon and nitrogen metabolism in *B. melitensis*.

## Results

### The *Brucella melitensis* 16M genome encodes four PTS proteins and HPr kinase/phosphorylase

The genome of *Brucella melitensis* 16M [22] contains three genes (*ptsP*/BMEI0190, *ptsO*/BMEI2031 and *ptsN*/BMEI1786) encoding homologues of the proteins composing the PTS^Ntr^ (EI^Ntr^, NPr and EIIA^Ntr^, respectively) and *ptsM*/BMEI2032 encoding a homologue of an EIIA of the mannose PTS family (EIIA^Man^-like). A homologue of HprK/P (encoded by *hprK*/BMEI2034) is also found in *Brucella*. All these genes are highly conserved in the genome of other sequenced *Brucella* species. Sequence analyses and multiple aligments of these five proteins were carried out and allowed the prediction of the phosphorylatable histidine or serine residues (see Figures S1 to S5). Similar to its homologues in *A. tumefaciens* and *S. meliloti*, *B. melitensis* EI^Ntr^ contains a GAF domain resembling the sensory domain of the NifA protein of *A. vinelandii* [6]. The GAF domains are ubiquitous motifs present in many sensory proteins of eukaryotes and prokaryotes and are proposed to allosterically regulate catalytic activities of these proteins through the binding of small molecules [13]. When compared to HprK/Ps from firmicutes, HprK/Ps from α-proteobacteria lack about 130 N-terminal amino acids. The role of this domain is still unknown and artificially truncated *L. casei* HprK/P (missing the first 127 amino acids) retained kinase and phosphorylase activities and all known regulatory properties [41]. Moreover, a carboxy-terminal conserved region is present in HprK/P from firmicutes and most β-, γ- and δ-proteobacteria, but absent from α-proteobacteria. This region was shown to be important for the phosphorylase activity of HprK/P, suggesting that in α-proteobacteria HprK/P might not be able to efficiently dephosphorylate P-Ser-NPr or dephosphorylate it by a different enzyme [30], [42].

Based on the sequence analyses, we propose that EI^Ntr^ autophosphorylates on His357 in the presence of PEP and subsequently transfers its phosphoryl group to His30 of NPr, which then phosphorylates EIIA^Ntr^ and the EIIA^Man^-like protein on His66 and His9, respectively. Moreover, we suggest that HprK/P might phosphorylate NPr on the conserved Ser61. These hypotheses were tested by carrying out *in vitro* phosphorylation assays with purified proteins.

### Phosphoryl group transfer from P∼EI to the EIIAs via P∼His-NPr

**Figure 1:**
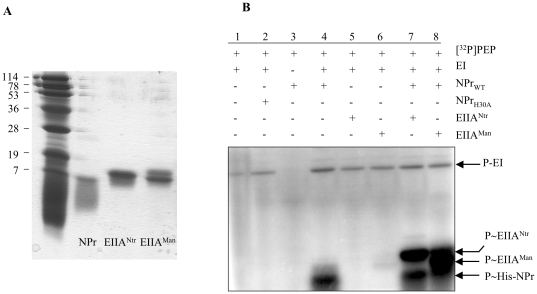
Purification and PEP-dependent phosphorylation of *B. melitensis* PTS proteins. His-tagged proteins were purified as described in Materials and Methods and analyzed on 0.1% SDS-15% polyacrylamide gels before carrying out phosphorylation experiments. (A) Electrophoretic separation of MW standards, NPr, EIIA^Ntr^ and the EIIA^Man^-like protein on a SDS gel stained with Coomassie Blue. (B) To carry out phosphorylation experiments, samples containing 10 µM [^32^P]PEP and the indicated proteins were incubated for 20 min at 37°C before they were separated on a 0.1% SDS-15% polyacrylamide gel, which was dried and exposed to a storage phosphor screen (see Materials and Methods). Arrows indicate the migration positions of EI^Ntr^, EIIA^Ntr^, EIIA^Man^-like and NPr/NPrH30A. Preparations of the EIIA^Man^-like protein always gave two bands migrating to nearly identical positions on SDS polyacrylamide gels and both became phosphorylated.

To carry out *in vitro* phosphorylation assays the *ptsP*, *ptsN*, *ptsM* and *ptsO* genes as well as a mutated *ptsO* allele (*ptsO*H30A) causing a His30Ala replacement in NPr, were inserted into a His_6_ tag expression vector and the resulting fusion proteins were purified as described in Materials and Methods (see also Fig. 1A for the purification of NPr, EIIA^Ntr^ and EIIA^Man^-like). We first tested the ability of EI^Ntr^ to phosphorylate NPr on His30 in a PEP-dependent reaction. NPr was not phosphorylated when incubated with [^32^P]PEP (Fig. 1B, lane 3). In agreement with our prediction (Fig. S4), the additional presence of EI allowed the phosphorylation of wild-type NPr (Fig. 1B, lane 4) and NPr_S61A_ (data not shown), but not of NPr_H30A_ (Fig. 1B, lane 2). We subsequently tested whether *B. melitensis* EIIA^Ntr^ and the EIIA^Man^-like protein were phosphorylated by P∼His-NPr. Incubation of EIIA^Ntr^ or the EIIA^Man^-like protein with [^32^P]PEP (data not shown) or [^32^P]PEP and EI^Ntr^ (Fig. 1B, lanes 5 and 6) did not allow their phosphorylation. By contrast, EIIA^Ntr^ and the EIIA^Man^-like protein were phosphorylated by [^32^P]PEP in the presence of both general PTS proteins EI^Ntr^ and NPr, establishing that after its own phosphorylation on His30 by EI^Ntr^, P∼His-NPr is able to transfer its phosphoryl group to EIIA^Ntr^ and the EIIA^Man^-like protein (Fig. 1B, lanes 7 and 8). Purification of the EIIA^Man^-like protein provided always two distinct forms migrating to slightly different positions on SDS polyacrylamide gels (Fig. 1A), which apparently became both phosphorylated by P∼His-NPr. The reason for the appearance of two EIIA^Man^-like forms is not known, but a similar observation has been reported for EIIAB^Man^ from *Streptococcus salivarius*, which was also isolated in two distinct forms migrating to slightly different positions on SDS polyacrylamide gels [43].

### Serine 61 of NPr is the target of ATP-dependent phosphorylation catalyzed by HprK/P

**Figure 2 pone-0012679-g002:**
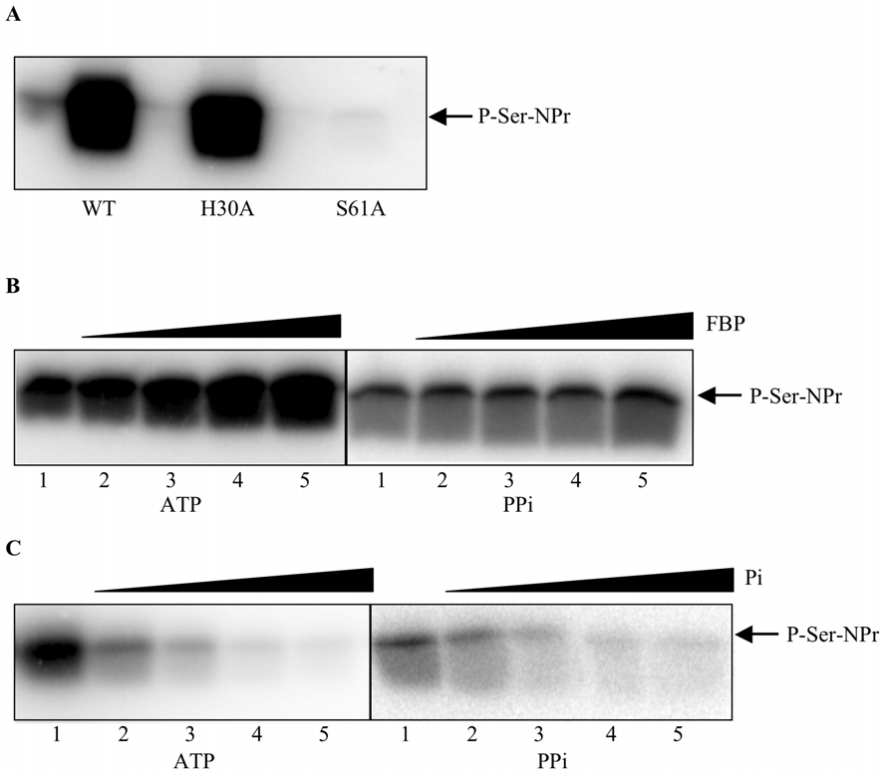
NPr kinase assays with *B. melitensis* HprK/P. (A) The NPr kinase assay was carried out with 200 ng of HprK/P and 2 **µ**g of either wild-type NPr (WT), NPr_H30A_ (H30A) or NPr_S61A_ (S61A) in the presence of 25 µM [γ-^32^P]ATP and in the absence of FBP and potassium phosphate (KPi). (B) Kinase assay with 200 ng of HprK/P and 3 µg of wild-type NPr, in the presence of 0.5 mM KPi, 25 µM [γ-^32^P]ATP or [^32^P]PPi and increasing concentrations of FBP (0, 1, 2.5, 5, 10 mM, lanes 1 to 5). (C) Kinase assay with 200 ng of HprK/P and 3 µg of wild-type NPr in the presence of 25 µM [γ-^32^P]ATP or [^32^P]PPi and increasing concentrations of potassium phosphate (0, 0.2, 1, 5, 25 mM, lanes 1 to 5).

The truncated HprK/P possibly adds an additional dimension of regulation to the *B. melitensis* PTS by phosphorylating NPr on a conserved serine residue (Ser61; see Fig S4). We therefore tested the ability of HprK/P to phosphorylate NPr on Ser61. As for the *pts* genes, we cloned the *hprK*-coding sequence in an expression vector and purified the His_6_-tagged fusion protein. As shown in Fig. 2A, HprK/P phosphorylated wild type NPr and NPr_H30A_ in an ATP-dependent way, whereas NPr_S61A_ was not phosphorylated.

### HPr kinase and P-Ser-HPr phosphorylase activities of HprK/P from *B. melitensis* 16 M

HprK/Ps of firmicutes possess antagonistic kinase (HPr phosphorylation) and phosphorylase (P-Ser-HPr dephosphorylation) activities, which are regulated by intracellular concentrations of inorganic phosphate (Pi) and glycolytic intermediates, such as fructose-1,6-bisphosphate (FBP) [28], [29], [44]. Indeed, the ATP-dependent kinase activity of HprK/P from *B. subtilis* is stimulated by FBP, but inhibited by Pi, which is also one of the substrates in the phosphorylase reaction. Moreover, in addition to ATP, HprK/P can also use pyrophosphate (PPi), the product of the HprK/P-catalyzed phosphorylase reaction, as phosphate donor [45]. The effect of increasing concentrations of FBP on ATP- and PPi-dependent kinase activities of *B. melitensis* HprK/P was tested. With both phosphoryl group donors, HprK/P was active as a kinase in the absence of FBP. Moreover, FBP has no stimulatory effect on the kinase activity in the absence of Pi (data not shown). Nevertheless, in the presence of 0.5 mM Pi, increasing concentrations of FBP (up to 10 mM) enhanced the ATP-dependent kinase activity, whereas under the same conditions almost no stimulatory effect was observed on the PPi-dependent activity (Fig. 2B). Similar results have been reported for *L. casei* HprK/P [45]. Kinase activity assays were also carried out in the presence of increasing concentrations of Pi. As observed for all studied Gram-positive HprK/Ps, the addition of Pi resulted in an inhibition of both the ATP- and PPi-dependent kinase activities of *B. melitensis* HprK/P (Fig. 2C).

**Figure 3 pone-0012679-g003:**
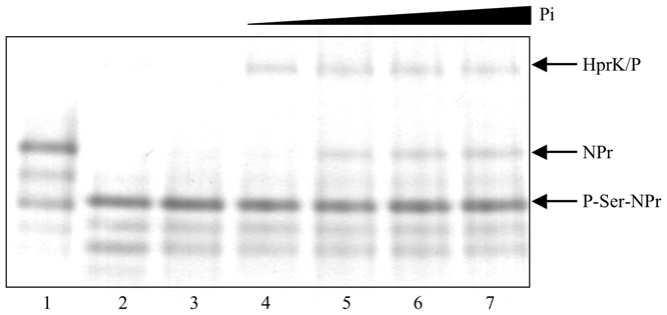
P-Ser-NPr dephosphorylation assay with *B. melitensis* HprK/P. Phosphorylase assays were carried out with 3 µg of P-Ser-NPr, 450 ng of HprK/P and increasing concentrations of KPi (2, 5, 10 and 20 mM, lanes 4 to7). Lane 1, 3 µg of NPr. Lane 2, phosphorylase assay with 100 ng HprK/P and no Pi. Lane 3, 3 µg of P-Ser-NPr.

We tested whether *B. melitensis* HprK/P also exhibits Pi-requiring phosphorylase activity. Even at high Pi concentrations (25 mM), P∼Ser-NPr was barely dephosphorylated by *B. melitensis* HprK/P (Fig. 3). In HprK/P of firmicutes, Pi binds to the same site as PPi and the β-phosphate of ATP, which is thought to be responsible for the inhibition of the ATP- and PPi-dependent kinase functions. However, although *B. melitensis* HprK/P seems to bind Pi, because its ATP- and PPi-dependent kinase activities are inhibited by Pi (Fig. 2C), *B. melitensis* HprK/P failed to promote efficient P∼Ser-NPr dephosphorylation. This seems to be the case for HprK/P from other proteobacteria, such as *A. tumefaciens* (I. Mijakovic, A. Khemiri and J. Deutscher, unpublished) and *Neisseria meningitidis* (S. Poncet, M.-K. Taha, M. Larribe and J. Deutscher, unpublished).

### P∼EIIA^Ntr^ is formed in an *hprK* mutant, but not in *pts* mutants or wild-type *B. melitensis*

**Figure 4:**
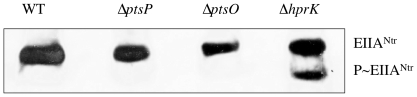
Detection of EIIA^Ntr^ and P*∼*EIIA^Ntr^ by Western blot in wild-type strain and Δ*pts* and Δ*hprK* mutants. Extracts from the wild-type, Δ*ptsP*, Δ*ptsO* and Δ*hprK* strains grown in 2YT (exponential phase; OD_600_ about 0.8) were loaded on non-denaturing polyacrylamide gels and subsequent electrophoresis allowed the separation of phospho and dephospho EIIA^Ntr^ (independently established with purified proteins; data not shown) and the corresponding bands of the two EIIA^Ntr^ forms were subsequently detected by Western blot with the anti-EIIA^Ntr^ polyclonal antibody. Identical results were obtained in a second independent experiment.

To ascertain that the PTS phosphorylation cascade is also functional *in vivo* we used Western blots in order to demonstrate the presence of P∼EIIA^Ntr^ *in B. melitensis* crude extracts. This was possible because we demonstrated with purified proteins that EIIA^Ntr^ and P∼EIIA^Ntr^ can be separated on non-denaturing polyacrylamide gels, with P∼EIIA^Ntr^ migrating significantly faster than EIIA^Ntr^ (data not shown). Extracts were prepared from the wild-type strain and the Δ*ptsP*, Δ*ptsO* and Δ*hprK* mutants grown in rich medium to exponential phase (OD_600_ = 0.8). and aliquots containing 60 µg of protein were loaded on a non-denaturing polyacrylamide gel. EIIA^Ntr^ and P∼EIIA^Ntr^ were separated by electrophoresis and detected by Western blotting with anti-EIIA^Ntr^ polyclonal antibodies. Under the conditions employed, only the slower migrating EIIA^Ntr^ band could be detected in extracts of the wild-type strain and the Δ*ptsP* and Δ*ptsO* mutants (Fig. 4). However, in the Δ*hprK* mutant an additional faster migrating band corresponding to P∼EIIA^Ntr^ was present. The absence of P-Ser-NPr, which is probably a poor substrate for the PEP-dependent phosphorylation, apparently allows significant phosphoryl transfer to EIIA^Ntr^.

### The PTS of *B. melitensis* is transcriptionally linked to the BvrR/S two component system

**Figure 5:**
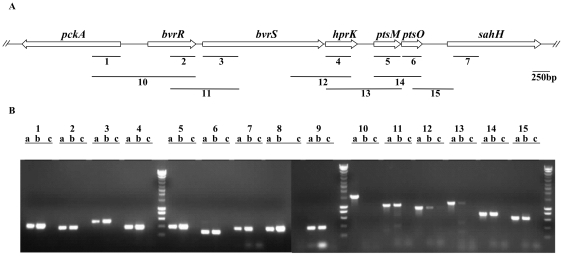
Transcriptional link between *pts* genes and the genes encoding the two-component system BvrR/BvrS. (A) Schematic representation of the genomic region encoding *hprK*, *ptsM* and *ptsO* in *B. melitensis* 16M. The regions amplified (1–15) by RT-PCR are indicated and the primers are listed in Table S2. (B) Agarose gel of the RT-PCR amplified products. For each primer pair, three lanes are shown: a, positive control using *B. melitensis*16M genomic DNA as template; b, RT-PCR; and c, a negative control using RNA as template (without RT). Identical results were obtained in several independent experiments.

The gene order around *B. melitensis hprK*, *ptsM* and *ptsO* is conserved in other *α*-proteobacteria and is as follows: (i) a transcriptional response regulator and (ii) a sensor kinase of a two-component system known to be involved in host-symbiont (*chvI*-*exoS* in *S. meliloti* [34]) or host-pathogen interaction (*chvI*-*chvG* in *A. tumefaciens* [33]; *bvrR-bvrS* in *B. abortus* [35]), (iii) *hprK*, (iv) *ptsM*, (v) *ptsO*, and finally (vi) *sahH*, which encodes an enzyme involved in the biosynthesis of methionine [36] (Fig. 5A). An additional gene called *pckA* that encodes PEP carboxykinase, a key enzyme of gluconeogenesis [39], [40] is oriented in opposite direction to this cluster (Fig. 5A). In order to see whether this conserved organization reflects a functional link between these genes, we tried to determine whether they were transcriptionally linked. For that purpose, we performed PCR assays using cDNA of *B. melitensis* 16M as template (Fig. 5B). Positive and negative control experiments were performed by using as template either genomic DNA or DNase-treated RNA in the absence of reverse transcriptase, respectively. When using cDNA as template we camplified intragenic regions of each gene of the cluster (Fig. 5A and B, bars and lanes 2 to 7), confirming that these genes are expressed in cells tat have grown in rich medium to late exponential phase. PCR products were also obtained for the intragenic regions of *ptsP* and *ptsN* (Fig. 5B, lanes 8 and 9, respectively), and for the neibhouring *pckA* gene (Fig. 5A and B, bar and lanes 1). The use of appropriate primers and cDNA as template also allowed the amplification of intergenic regions (lanes 11 to 15 in Fig. 5B), demonstrating that the following pairs of genes are co-transcribed: *bvrR-bvrS*, *bvrS-hprK*, *hprK-ptsM*, *ptsM-ptsO* and *ptsO-sahH* (Fig. 5B). As expected we could not amplify by RT-PCR the intergenic region between *pckA* and *bvrR*, two genes oriented in opposite directions (Fig. 5A and B, bar and lanes 10). In conclusion, we demonstrated that the *B. melitensis pts* genes and *hprK* are expressed during vegetative growth and that *hprK*, *ptsM* and *ptsO* can be co-transcribed with *bvrR/S* and *sahH*.

### Δ*ptsP* and Δ*ptsO* mutants barely produce VirB5 and VirB10

Knowing that transpositional PTS mutants of *B. melitensis* are attenuated [19], [20], [21] and having demonstrated a transcriptional link with several *pts* genes and the genes for the BvrR/S two component system, which regulates major virulence determinants [35], we wanted to investigate a possible link between PTS and virulence by constructing deletion mutants of the corresponding genes. Since *hprK*, *ptsM* and *ptsO* are probably organized in an operon with *bvrR, bvrS* and *sahH*, we chose to construct the mutants by allelic replacement using the non-polar cassette *aphA4* as previously described [46]. Mutants were obtained for the *ptsP*, *ptsO*, *ptsN* and *hprK* genes. Despite numerous attempts, we were not able to delete *ptsM*.

**Figure 6:**
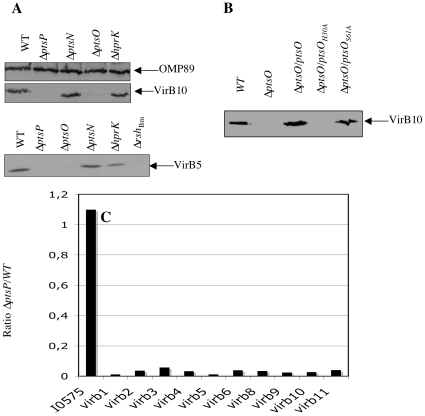
Synthesis of VirB proteins in Δ*hprK* and Δ*pts* mutants. (A) Detection of VirB10 (top) and VirB5 (bottom) by Western blot analysis in the wild-type (*Bm*16M), Δ*ptsP*, Δ*ptsO*, Δ*ptsN* and Δ*hprK* strains following growth in 2YT to late-exponential phase. An established negative control (Δ*rsh_Bm_*) was included in the anti-VirB5 Western blot analysis [46] (B) Western blot analysis of VirB10 with *Bm*16M (+pMR10*cat*), Δ*ptsO* (+pMR10*cat*) and the complemented strain Δ*ptsO/ptsO*, Δ*ptsO/ptsO_H30A_*, *and* Δ*ptsO/ptsO_S61A_*. Identical results were obtained in a second independent experiment. (C) Transcription analysis of *virB* gene expression in wild-type and *ptsP* mutant. The values presented by the bars correspond to the ratio of normalized and averaged microarray data (n = 2×3) obtained for 10 *virB* ORFs in the *ptsP* mutant and the wild-type strain grown under the same conditions. BMEI0575 is a control ORF whose expression is not modulated whatever the strain considered.

**Figure 7:**
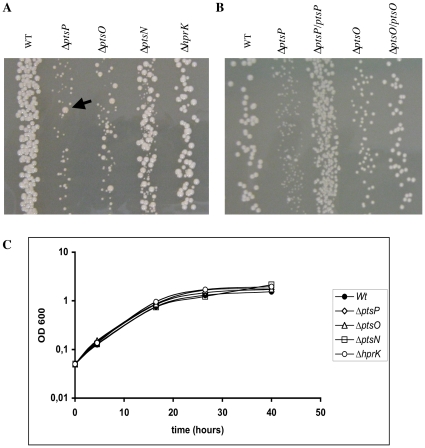
Δ*pts* mutants mutants display colony size heterogeneity on solid medium. (A) Colony size heterogeneity displayed by the mutants Δ*ptsP*, Δ*ptsO*, Δ*ptsN* and Δ*hprK* in comparison to the wild-type strain. Late log phase cultures were diluted and plated on 2YT medium and grown at 37°C for 8 to 10 days. (B) Complementation of the colony size heterogeneity phenotype for the Δ*ptsP* and Δ*ptsO* mutants. Wild-type, Δ*ptsP* and Δ*ptsO* strains (carrying the empty vector pMR10-*cat*; see Table S1) and the complemented mutants Δ*ptsP/ptsP* and Δ*ptsO/ptsO* (carrying vectors pRH001-*ptsP* and -*ptsO*, respectively; see Table S1) were plated on 2YT supplemented with 20 µg/ml of chloramphenicol as described in (A) and grown at 37°C for 8 to 10 days. (C) Growth of Δ*pts* and Δ*hprK* mutants in 2YT liquid cultures in comparison to the wild-type strain. Overnight 2YT cultures of the five strains were back diluted to an OD_600_ (optical density at 600 nm) of 0.05, and growth was monitored by measuring the OD_600_ at diffent time intervals. Identical results were obtained in three independent experiments. When re-isolated on new plates small and large colonies give always the same size of colonies (except for some “suppressors” that appeared in the small phenotype background (see arrowhead in figure 7A).

VirB is a major virulence factor of *Brucella* spp. composed of twelve subunits encoded in the *virB* operon [47], [48], [49], [50], that is induced in response to nutrient availability [46], [51], [52] and controlled by (p)ppGpp, a bacterial alarmone that mediates global physiological control in response to starvation [46]. Since PTS proteins are also involved in global regulation in response to nutrient supply (for review see [1]), we examined the role of *pts* genes and *hprK* in the control of *virB* expression. Western blot analyses using anti-VirB5 and anti-VirB10 antisera [53] were performed to determine the relative amounts of VirB5 and VirB10 proteins in Δ*ptsP*, Δ*ptsO*, Δ*ptsN* and Δ*hprK* mutants compared to the wild-type strain *B. melitensis* 16M. Crude extracts of bacteria grown in 2YT to late exponential – early stationary phase (OD_600_ of 0.8–1.2) were prepared and analyzed by Western blot. The Δ*ptsN* and Δ*hprK* mutants produced VirB5 and VirB10 in amounts similar to those of the wild-type strain (Fig. 6A). However, no or very little VirB5 and VirB10 were detected in extracts prepared from Δ*ptsP* and Δ*ptsO* mutants, suggesting that EI^Ntr^ and NPr are required for production or stability of several *B. melitensis* VirB subunits (Fig. 6A). Complementation of the Δ*ptsO* mutant with wild-type *ptsO* constitutively expressed from a low copy plasmid fully restored VirB10 (Fig. 6B) and VirB5 production (data not shown). For unknown reasons, plasmid-encoded *ptsP* did not restore VirB5 or VirB10 production in the Δ*ptsP* mutant, although the plasmid was functional because it complemented the “small colony” phenotype of the Δ*ptsP* mutant (see Fig. 7B). Knowing that EI**^Nt^** ^***r***^ is strictly required for P∼His-NPr formation we tried to complement the Δ*ptsO* mutant with the mutant alleles *ptsO_H30A_* and *ptsO_S61A_*. Constitutive expression of *ptsO* and *ptsO_S61A_* in Δ*ptsO* restored VirB10 production, whereas the ΔptsO/*ptsO_H30A_* strain failed to produce VirB10 (Fig. 6B). This confirms that P∼His-NPr is needed for VirB 10 synthesis and consequently that EI^Ntr^ is also required.

In order to confirm the impact of the *ptsP* mutations on T4SS expression we carried out a transcriptional analysis of the whole *virB* operon with the wild-type strain and the *ptsP* mutant. Indeed, the expression level of the individual *virB* genes was more than thirty times lower in the *ptsP* mutant than in the wild-type strain (Fig. 6C)

### Colony size heterogeneity of *pts* and *hprK* deletion mutants plated on rich medium

When plated on 2YT rich medium, the Δ*ptsP*, Δ*ptsO*, Δ*ptsN* and, to a lesser extent, the Δ*hprK* mutant displayed a heterogeneity in colony size compared to the wild-type strain *B. melitensis* 16M (Fig. 7A). Small colonies were detected only 8 to 10 days after inoculation, whereas the larger colonies were visible after 3 to 4 days as usually observed for the wild-type strain. To ensure that the colony size heterogeneity of *pts* and *hprK* mutants resulted from the deletion of the corresponding genes, we first carried out a complete typing of these strains confirming that they all derived from *B. melitensis* 16M wild-type, exhibited a smooth phenotype, and were not contaminated with other strains (data not shown). Next, we complemented the two mutants with the most marked phenotype (Δ*ptsP* and Δ*ptsO*) by constitutively expressing wild-type copies of the *ptsP* and *ptsO* genes in the corresponding mutants. As shown in Fig. 7B, the complemented strains Δ*ptsP/ptsP* and Δ*ptsO/ptsO* displayed bigger colonies than the Δ*ptsP* and Δ*ptsO* mutants transformed with the empty vector-pMR10*cat*. Their colonies resembled those of the wild-type strain carrying the empty vector-pMR10*cat*.

Finally, we measured growth of the four mutants when cultivated in liquid 2YT medium (Fig. 7C). No differences were observed between growth of the mutants and the wild-type strain, suggesting that the growth heterogeneity observed on solid medium might not result from the composition of the medium, but rather from parameters that distinguish liquid and solid cultures, such as oxygen supply, nutrient or water availability.

### Yeast two hybrid assays reveal oligomerization of EI^Ntr^, the EIIA^Man^-like protein and HprK/P, and interaction between NPr and HprK/P

**Figure 8:**
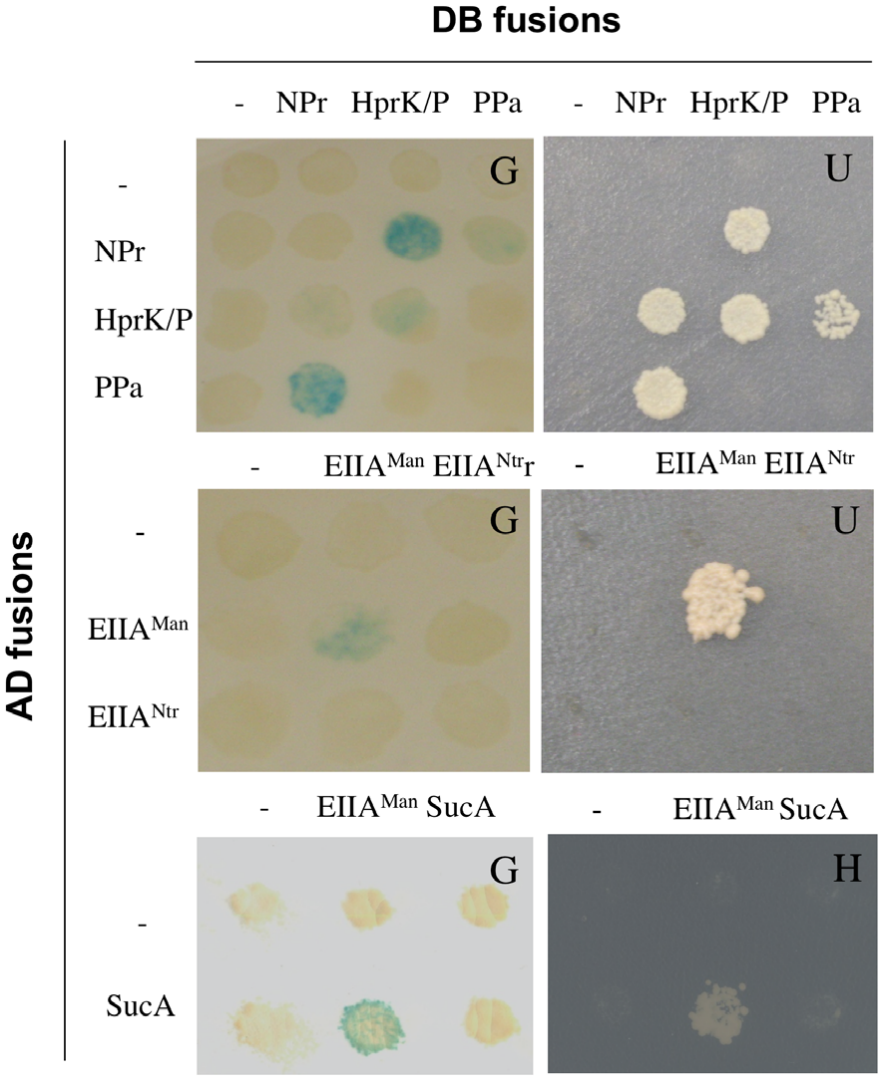
Detection of interaction partners for *Brucella* PTS proteins by Y2H assays. Interaction between NPr, HprK/P and PPa (top row). Interaction between EIIA^Man^-like and EIIA^Ntr^ (middle row). Interaction between EIIA^Man^-like and SucA (bottom row). AD fusion  =  protein of interest fused with activating domain of Gal4; BD fusion  =  protein of interest fused with DNA binding domain of Gal4. The reporter used is indicated by the letter in the upper right corner of each picture: β-galactosidase activity (G); growth test without uracil (U); growth test without histidine in the presence of 40 mM of 3AT (H). “-” indicates an empty vector. Identical results were obtained in three independent experiments.

Since in *B. melitensis* 16M two *pts* genes, *hprK* and the two-component system genes *bvrR/bvrS* are co-transcribed and functionally linked we wanted to test if there existed any physical interactions between PTS components, HprK/P and the BvrR/S proteins. A yeast two hybrid (Y2H) interaction matrix of 64 interactions was performed with the four PTS proteins, HprK/P, BvrR and BvrS fused to the Gal4 DNA binding domain (BD) and tested against the same proteins fused to the Gal4 activating domain (AD). Each BD and AD fusion was also tested against Gal4-AD and Gal4-BD alone. The previously evidenced interaction between BvrR and BvrS [54] was used as a positive control. The results presented in Fig. 8 and S6 show that two PTS proteins (EI^Ntr^, EIIA^Man^-like) and HprK/P interacted with themselve, suggesting that these proteins form oligomers similar to some well-studied EI, EIIA^Man^ and HprK/P homologues [55], [56], [57]. Additionally, a bidirectional interaction was evidenced between NPr and HprK/P (Fig. 8 and S6) confirming the results of the *in vitro* phosphorylation test. No interaction was observed between other PTS proteins that were shown to phosphorylate each other *in vitro*. Finally, an interaction between BvrR and BvrS could be demonstrated (Fig. S6), but no interaction was detected between any of the PTS proteins or HprK/P and the two-component partners.

### A yeast two-hybrid screen against the *B. melitensis* ORFeome reveals interaction partners of the EIIA^Man^-like protein and NPr

Having demonstrated that the incomplete PTS of *B. melitensis* is functional and knowing that PTS-dependent regulations are mediated either by allosteric interaction or by direct phosphorylation of target proteins [1], we performed a Y2H screen to detect interaction partners of the EIIA^Man^-like protein and NPr of *B. melitensis* 16M to get some preliminary clues about the functional role of this PTS. Briefly, these PTS proteins were fused to the Gal4 DNA binding domain and used as baits to identify interaction partners in an « ORFeomic » library. Two clones provided a positive signal with at least two of the three reporter genes and the corresponding proteins were identified as Ppa and SucA by sequencing the inserts in the pVV213 vector. NPr interacts with the inorganic pyrophosphatase PPa (BMEI0076) and the EIIA^Man^-like protein with the E1 component (SucA) (BMEI0140) of 2-oxoglutarate dehydrogenase.

In order to validate these interactions, the ORFeome entry clones for *ptsO* (NPr), *ppa*, *hprK*, *sucA* and *ptsM* (EIIA^Man^-like) were checked by sequencing and the coding sequences were subcloned in the Y2H vectors pVV212 and pVV213. Three interaction matrices were designed and the interactions between NPr and Ppa and the EIIA^Man^-like protein and SucA were confirmed (Fig. 8). In addition, a new interaction between PPa and HprK was established. SucA is the E1 component of the 2-oxoglutarate dehydrogenase complex, which contains also the dihydrolipoamide succinyltransferase SucB (E2 component) and dihydrolipoamide dehydrogenase (E3 component) and plays a crucial role in the TCA cycle by converting 2-oxoglutarate to succinyl-CoA and CO_2_. Knowing that the PTS^Ntr^ presumably links regulation of carbon and nitrogen metabolism [1], [2] and that 2-oxoglutarate is at the cross-road between the TCA cycle and nitrogen assimilation, we tried to confirm the interaction between the EIIA^Man^-like protein and SucA by another independent method.

### SucA, the E1 component of the enzymatic 2-oxoglutarate dehydrogenase complex physically interacts with the EIIA^Man^-like protein

DivIVA is attracted to and remains at cell poles not only in its native organism, *B. subtilis*, but also in *E. coli* and other bacteria [58]. In addition, DivIVA fused to a “bait” protein X can target an interacting GFP tagged “prey” protein Y to the pole [59]. To confirm the interaction between the EIIA^Man^-like protein and SucA we fused DivIVA to SucA and the EIIA^Man^-like protein to GFP.

**Figure 9:**
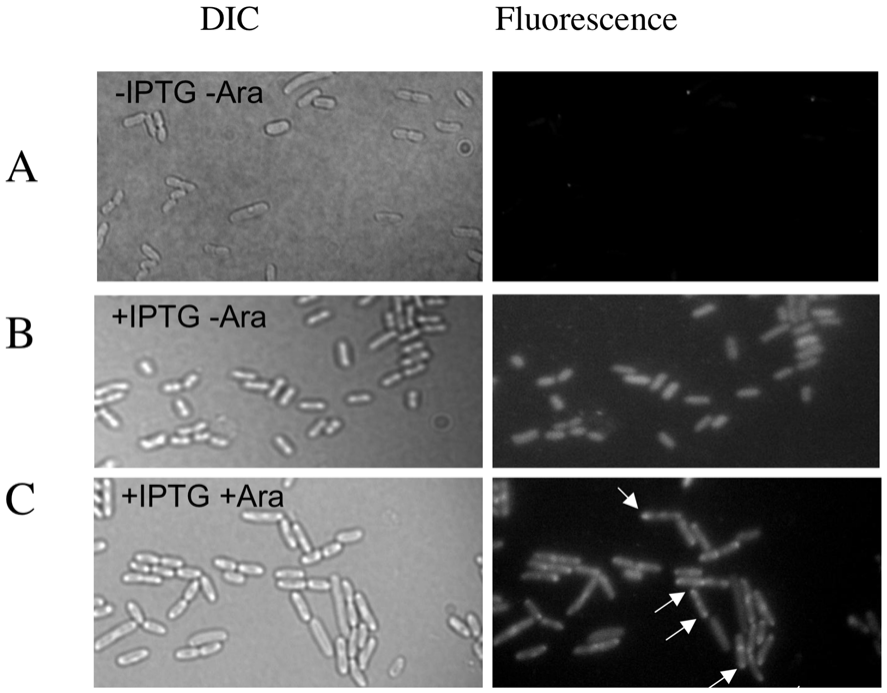
The EIIA^Man^-like protein interacts with SucA. DIC and corresponding fluorescent images were taken from *E.coli* (pSK*ori*T*cat* –pBad-*divIVA-sucA* and pMR10*kan-ptsM-gfp*) grown in different conditions: (A) without IPTG and arabinose (no synthesis of EIIA^Man^-GFP and SucA-DivIVA).(B) with IPTG and no arabinose (synthesis of EIIA^Man^-GFP) and (C) with both IPTG and arabinose (EIIA^Man^-GFP and SucA-DivIVA are synthesized). Only in the latter case EIIA^Man^-GFP co-localizes with SucA-DivIVA at the cell poles.

After arabinose induction, strain DH10B[pSK*ori*T*cat*-pBad-*divIVA-gfp*] synthesizing DivIVA-GFP exhibits fluorescens mainly at the cell poles (positive control; data not shown), whereas DH10B[pMR10*kan*-*ptsM*-*gfp*] producing EIIA^Man^-GFP (with or without arabinose induction) was uniformly fluorescent (Fig. 9B; negative control). DH10B bearing the two plasmids (pSK*ori*T*cat*-pBad-*divIVA-sucA* and pMR10*kan*-*ptsM*-*gfp*) showed a bipolar fluorescence pattern only when arabinose was present (Fig. 9C). This illustrates that SucA was targeted to the pole by DivIVA and able to recruit the EIIA^Man^-GFP fusion at the same location, thus confirming the interaction detected in the Y2H experiments.

## Discussion

**Figure 10:**
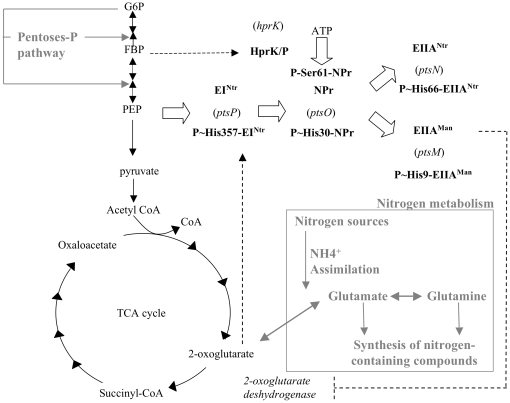
Model proposed for the role of the *Brucella* PTS in connecting C and N metabolisms. In agreement with the results of the *in vitro* and *in vivo* phosphorylation assays (Fig. 1 [Fig pone-0012679-g002] [Fig pone-0012679-g003]–4), we postulate that a phosphoryl group is sequentially transferred from PEP to EI^Ntr^, NPr, and finally to the EIIA^Man^-like protein. By binding both an unknown ligand (possibly 2-oxoglutarate) through its GAF domain and autophosphorylating in response to the PEP/pyruvate ratio, EI^Ntr^ might sense the metabolic status of the cell and communicate it to the EIIA^Man^-like protein that would regulate the 2-oxoglutarate dehydrogenase activity accordingly. In addition, HprK/P is expected to slow phosphorylation of the EIIA^Man^-like protein by hindering the phosphotransfer through the PTS in response to changes in the FBP concentration or other metabolites. Solid arrows indicate metabolic reactions, large open arrows represent phosphoryl transfer between PTS proteins, and dashed arrows show putative regulatory processes. The enzyme HprK/P was also included in this scheme since it phosphorylates NPr on a conserved serine. The names of the genes encoding *B. melitensis* PTS and HprK/P proteins are put between brackets. The pentose phosphate pathway, which connects glucose-6-phosphate (G6P) to glycolysis in *B. melitensis*, and the connection between TCA cycle and nitrogen metabolism starting from 2-oxoglutarate are indicated in grey. FBP, fructose-1,6-bisphosphate; PEP, phosphoenolpyruvate; CoA, coenzyme A; TCA cycle, tricarboxylic acid cycle; NH4^+^, ammonium. P∼H-X and P∼S-X indicate PTS enzymes phosphorylated on histidine or serine, respectively.

Since PTS permeases are lacking in α-proteobacteria, their soluble PTS proteins are not involved in carbohydrate transport and phosphorylation, but probably participate only in a regulatory phosphorelay (Fig. 10) [25], [26], [27]. In this paper, we present the first extensive biochemical and genetic characterization of the PTS components in an organism that lacks PTS permeases. A few studies on similar systems have previously been carried out, but were either limited to biochemical studies of PTS protein phosphorylation [60] or to genetic studies of mutants [61]. First we established that in *B. melitensis* the phosphoryl group transfer from PEP to the EIIAs is fully functional. Second, several pieces of evidence allow us to propose a link between the PTS and the virulence of *B. melitensis*. Finally, we report a connection between the PTS and systems likely to maintain the N/C balance. These three points are discussed in detail hereunder.

### 1- The PTS phosphorelay of *B. melitensis* is fully functional and senses the metabolic state

*B. melitensis* EI^Ntr^ autophosphorylates with PEP and transfers the phosphoryl group to the conserved His30 of NPr before it is passed on to either EIIA^Ntr^ or the EIIA^Man^-like protein (Fig. 10). EI^Ntr^ probably senses the PEP availability (PEP/pyruvate ratio), that is translated into relative levels of phosphorylated vs. non-phosphorylated forms of NPr and EIIAs [62]. In addition to His30, NPr is also phosphorylated on a serine residue. Similar to firmicutes, *B. melitensis* possesses an HprK/P using ATP or PPi as phosphoryl donor. Only phosphorylation of NPr with ATP is stimulated by FBP (Fig. 2B). Identical observations were made for *L. casei* HprK/P [45]. However, *Brucella spp* lack a 6-phosphofructokinase and consequently hexose catabolism does not occur via the Embden-Meyerhof-Parnas pathway, but is redirected through the pentose phosphate and perhaps the Entner-Doudorof pathway. Accordingly, the only pathway that is expected to produce FBP is gluconeogenesis [63]. We therefore propose that, in contrast to firmicutes, the FBP signal sensed by *B. melitensis* HprK/P reflects gluconeogenetic instead of glycolytic activity. High gluconeogenetic flux will probably activate HprK/P (via FBP) (Fig. 2B), which in turn will slow the PEP-dependent phosphoryl transfer from P-Ser-NPr to the EIIAs (Fig. 4) [1], [64], [65].

Interestingly, an inorganic pyrophosphatase named PPa interacts with NPr and HprK/P in Y2H tests (Fig. 8). PPi serves as substrate for the kinase reaction and is formed during P-Ser-HPr dephosphorylation [45]. Hydrolysis of PPi by PPa not only lowers the PPi concentration, but also produces inorganic phosphate (Pi), which inhibits both, ATP- and PPi-dependent kinase activities of *B. melitensis* HprK/P (Fig. 2C). Elevated PPa activity might therefore reduce phosphorylation on Ser61 of NPr. Similarly, Mijakovic [45] proposed that the *B. subtilis* pyrophosphatase YvoE (the *yvoE* gene is located in the *hprK* operon) indirectly decreases the kinase activity of HprK/P, and stimulates P-Ser-HPr dephosphorylation by HprK/P. The physical interaction of PPa with NPr and HprK/P might allow efficient regulation of HprK/P activity in *B. melitensis*. Alternatively, a link might exist between the PTS and the ppGpp production/degradation system (also called stringent response) as was demonstrated in *E. coli* [66]. NPr might affect the PPa-catalyzed conversion of PPi to Pi and thus modulate the PPi-producing ppGpp degrading activity of Rsh (RelA/SpoT homologue).

Purified *B. melitensis* HprK/P barely dephosphorylated P-Ser-NPr under *in vitro* conditions (Fig. 3). Similar observations were made for HprK/P from *Treponema denticola* [60], *M. pneumoniae* [67], *N. meningitidis* (S. Poncet, M.-K. Taha, M. Laribe and J. Deutscher, unpublished) and *A. tumefaciens* (I. Mijakovic, A. Khemiri and J. Deutscher, unpublished). In the case of α-proteobacteria, the poor phosphorylase activity of HprK/P might be due to the absence of a C-terminal conserved region required for P-Ser-HPr dephosphorylation (Fig. S5) [30], [41], [42]. In *M. pneumoniae* and possibly other bacteria, dephosphorylation of P-Ser-HPr seems to be catalyzed by a protein phosphatase of the PP2C family [67].

### 2- A link between PTS and virulence of *Brucella*

It seems that PTS-mediated carbon source utilization can affect host-bacteria interactions [61], [68]. *B. melitensis pts* mutants were previously shown to be attenuated [19], [20], [21] but the underlying mechanism remained unknown. During this work two lines of evidence for a link between PTS and virulence emerged. First, we demonstrate a transcriptional link between the PTS genes *hprK, ptsM* and *ptsO* and the *bvrR/S* genes (Fig. 5) encoding a two component system crucial for virulence of *B. melitensis*. In all pathogenic or symbiotic α-proteobacteria the *pts* genes are located downstream from the two-component system genes essential for infection or symbiosis [33], [34], [35]. In addition, a recent transcriptome analysis with *B. abortus* showed that *hprK* (BAB1_2094) was downregulated in the *bvrR:Tn5* mutant [69]. It is therefore tempting to assume that the PTS might also be involved in virulence regulation, possibly via a cross-talk between PTS proteins and the two-component system. This concept is also supported by the finding that deletion of *ptsP* or *ptsO*, but not *ptsN* or *hprK*, lowers the production of a major virulence factor, the type IV secretion system VirB, by reducing *virB* gene expression (Fig. 6). The expression of *Brucella* spp. *virB* has previously been shown to be controlled by nutrient availability via an unknown mechanism [46], [48], [51]. The *Bartonella henselae* BvrR/S homologues BatR/S, whose genes are also followed by *hprK*, *ptsM* and *npr*, have been reported to control *virB* expression and BatR binds to the *virB* promoter region [70]. It is therefore tempting to assume that the *B. melitensis* PTS communicates the metabolic state of the cell to the *virB* promoter by phosphorylating or interacting with the two component system BvrR/S. However, we cannot exclude the possibility that PTS components interact with one of the two other transcriptional regulators known to bind to the *virB* promoter: the quorum sensing regulator VjbR [52], [71] and HutC, a transcriptional repressor of the histidine utilization (*hut*) genes [72].

While no differences were observed between growth of the four mutants and the wild-type strain when cultivated in liquid 2YT medium (Fig. 7C), the Δ*ptsO*, Δ*ptsP* and to a lesser extent the Δ*ptsN* and Δ*hprK* mutants displayed a heterogeneity in colony size compared to the wild-type strain when plated on solid 2YT rich medium (Fig.7A and C). This is reminiscent of a similar defect described for *pts* mutants of *S. meliloti* grown on solid media [61]. These small colonies resemble a phenotype called small colony variant (SCV). The presence of SCVs in pathogenic bacteria, including *B. abortus*, has often been associated with the persistence in the host [73], [74], [75], [76], [77]. It will be interesting to test whether *pts* and *hprK* mutants persist longer than the wild-type during mice infection, as was described for a SCV of the *B. abortus* vaccinal strain S19 [73].

### 3- Carbon catabolite repression and the coordination of carbon and nitrogen metabolism

In many bacteria the PTS is linked to carbon catabolite repression (CCR). Interestingly, in the α-proteobacterium *S. meliloti* HprK/P regulates succinate-mediated CCR [32]. In *Brucella*, erythritol is the most favoured carbon source and is able to inhibit glucose incorporation [78], but to our knownledge the underlying mechanism is not known and diauxic growth has not been reported. CcpA as well as Crp [79] and adenylate cyclase seem to be absent from *Brucella* spp. If general CCR exists in *Brucella*, it should therefore differ from the *E. coli* and *B. subtilis* CCR mechanisms.

The PTS^Ntr^ proteins (EI^Ntr^, NPr and EIIA^Ntr^) have previously been suggested to provide a regulatory link between carbon and nitrogen metabolism [3], [4], [5], [6], [11], [80], [81]. Additionally, recent “*in silico*” analyses suggest that some of the diverse regulatory PTS functions acquired during evolution serve to assure an appropriate balance in C and N supply [82]. Key signals of C and N supply in *E. coli* appear to be the levels of glutamine and 2-oxoglutarate, the latter being at the crossroad between carbon and nitrogen metabolism [83]. Several results reported in our paper converge on these metabolites and prompted us to propose a model linking the PTS to the maintenance of the carbon and nitrogen balance in *B. melitensis* 16M:

First, the EIIA^Man^-like protein interacts with the SucA subunit of 2-oxoglutarate dehydrogenase (Fig. 8 and 9).
Second, the enzyme EI^Ntr^ possesses an N-terminal GAF domain (Fig. S1). This domain is known to regulate the activity of NifA from *A. vinelandii* by binding 2-oxoglutarate [84], [85].
Finally, three PTS genes are transcriptionaly linked to the genes encoding the two component system BvrR/S (Fig. 5).

The latter finding supports a link between regulation of C and N metabolism and PTS^Ntr^ components because a proteomic study with a *B. abortus bvrR* mutant [86] revealed that two 2-oxoglutarate-dependent proteins are regulated by BvrR-BvrS: the first is the PII sensor protein that controls nitrogen metabolism and that was shown to bind 2-oxoglutarate [83], [87]; the second is the 2-oxoglutarate dehydrogenase complex that converts 2-oxoglutarate into succinyl-CoA in the TCA cycle. This is the very same enzyme whose subunit SucA interacts with the EIIA^Man^-like protein (Fig. 8 and 9).

We therefore propose a model in which EI^Ntr^ senses the metabolic status of the cell via the PEP/pyruvate ratio [62]. The existence of a GAF domain in EI^Ntr^ provides a link between GAF-sensed signals (α-ketoglutarate [84], [85] or other ligands [88]) and PTS phosphoryl transfer. The signals (EI phosophorylation state and HprK/P activity) are transmitted to the EIIA^Man^-like protein, which in turn regulates the activity of 2-oxoglutarate dehydrogenase (Fig. 10). In this model, the dephospho EIIA^Man^-like protein is predicted to interact with and to inhibit 2-oxoglutarate dehydrogenase. Indeed, exclusively dephospho EIIA^Man^ is probably present in yeast during two-hybrid tests, where the EIIA^Man^/SucA interaction was first detetcted. Finally, one can envisage that HprK/P might control EIIA^Man^-dependent regulation of 2-oxoglutarate dehydrogenase. Similar as observed for HPr from firmicutes [1], [64], [65], HprK/P-catalyzed phosphorylation of Ser61 of NPr probably slows phosphorylation of His30 and thus increases the amount of dephospho EIIAs (Fig. 4).

It will also be interesting to test whether the N-terminal domain of EI^Ntr^ is able to bind 2-oxoglutarate and whether this ligand can modulate the phosphotransfer activity of the PTS protein. It might also be worth studying the enzymatic activity of 2-oxoglutarate dehydrogenase in different mutant backgrounds. Finally, our model should be tested with other α-proteobacteria possessing homologues of the PTS regulatory proteins and the crucial two component system encoded by genes arranged in a strictly conserved genomic context.

## Materials and Methods

### Ethics statement

Animal handling and experimental protocol was in accordance with European (DOCE 86/609/EEC), and National (AR25/04/2004) directives, and was supervised and authorized by the Ethical Committee of the University of Namur (FUNDP) (Commission d'éthique en experimentation animale approval N° FUNDP08/106).

### Bacterial strains and growth conditions

All *Brucella* strains used in this study were derived from *Brucella melitensis* 16M Nal^R^ (Table S1) and were routinely cultivated in 2YT complex medium (10% yeast extract, 1% tryptone and 0.5% NaCl). *E. coli* strains (Table S1) were cultivated in Luria Bertani (LB) medium. Antibiotics were used at the following concentrations when appropriate: nalidixic acid, 25 µg/ml; kanamycin, 50 µg/ml; chloramphenicol, 20 µg/ml; ampicillin, 100 µg/ml; gentamycin, 50 µg/ml.

To observe the colony size heterogeneity of *pts* and *hprK* mutants, overnight cultures were adjusted to an OD_600_ of 0.05 in 2YT complex medium and grown at 37°C with constant shaking until late log phase (OD_600_ of 1.0). Dilutions of these cultures were plated on 2YT agar supplemented with appropriate antibiotics and incubated for 8 to 10 days at 37°C.

To evaluate growth of the *pts* and *hprK* mutants in liquid cultures, overnight cultures were diluted to an OD_600_ of 0.05 in 2YT complex medium and grown at 37°C with constant shaking. The experiment was carried out twice.

### Construction of overexpression plasmids

For the construction of overexpression plasmids, the *B. melitensis* ORFeome entry vectors [89] bearing *ptsN, ptsM* and *hprK* (pDONR201-ptsN, ptsM and hprK, respectively – Table S1) were checked by DNA sequencing before they were used to amplify by PCR the *ptsN, ptsM* and *hprK* genes with oligonucleotide pairs SP67-SP68, SP69-SP70, and SP74-SP75 respectively (Table S2). The *ptsN*-, *ptsM*- and *hprK*-PCR products were digested with BamHI and KpnI and cloned into pQE30 (Table S1) digested with the same enzymes, resulting in plasmids pQE30-*ptsN*, -*ptsM*- and –*hprK* and encoding (His)_6_-EIIA^Ntr^, (His)_6_-EIIA^Man^-like and (His)_6_-HprK/P, respectively (Table S1). The correct sequence of all PCR products was confirmed by DNA sequencing.

The published genome sequence predicts a *ptsO* gene starting with a GUG codon and lacking a ribosome binding site (RBS) [22]. We therefore assumed that NPr might be 4 amino acids longer and that the gene starts with an ATG preceded by a RBS. Accordingly, a new pDONR201 entry vector (pDONR201-*ptsO*) bearing a longer version of the *ptsO* gene was constructed. Briefly, the *B. melitensis* 16M *ptsO* CDS (BMEI2031) was amplified by PCR with genomic DNA with and the Gateway™ primers GWnprF and GWnprR (Table S2) and cloned in the entry vector pDONR201 (Invitrogen Life-technologies) as previously described [89]. Directed mutagenesis of *ptsO* was performed with the QuickChange™ Site Directed Mutagenesis kit (Stratagene) using plasmid pDONR201-*ptsO* as a template. Primers used to obtain the *ptsO*H30A and *ptsO*S61A alleles are listed in Table S2. The correct sequence of all PCR products was confirmed by DNA sequencing. Plasmids pQE30-*ptsO*, -*ptsO*H30A and -*ptsO*S61A encoding (His)_6_-NPr and its two mutant forms, were obtained by amplification of the corresponding allele using oligonucleotides SP65 and SP66, and plasmids pDONR201-*ptsO*, -*ptsO*H30A and -*ptsO*S61A respectively, as templates. The PCR products were digested with *BamHI* and *KpnI* and cloned into pQE30 digested with the same enzymes.

### Overexpression and purification of PTS proteins

The *E. coli* NM522 (Stratagene) transformants (Table S1) harboring the various pQE30-derivatives were grown in 500 ml of LB medium supplemented with 100 µg/ml ampicillin to an OD_600_ of 0.7. The synthesis of (His)_6_-fusion proteins was induced with 0.1 mM isopropyl-β-D-thiogalactopyranoside and growth was continued for 3 hours at 37°C. Protein extracts were prepared and loaded on a 1 ml Ni-NTA column (Qiagen); purification was carried out under native conditions by following the recommendations of the manufacturer. (His)_6_-tagged EI^Ntr^, NPr, NPrH30A, NPrS61A, EIIA^Ntr^, EIIA^Man^-like and HprK/P were recovered as soluble proteins.

### Protein phosphorylation and dephosphorylation assays

[^32^P]PEP was synthesized by following the PEP-pyruvate isotope exchange method in the presence of pyruvate kinase and [**γ**-^32^P]ATP [90]. Transfer of the phosphoryl group from [^32^P]PEP via EI^Ntr^, NPr or NPrH30A to EIIA^Ntr^ or EIIA^Man^-like was tested at 37°C in 30 µl reaction mixtures containing 50 mM Tris-HCl pH 7.4, 5 mM MgCl_2_, 10 µM [^32^P]PEP, 1.5 µg of EI^Ntr^, 3 µg of NPr or NPrH30A, 4.5 µg of EIIA^Ntr^ or EIIA^Man^-like. Samples were incubated for 20 min at 37°C and reactions were stopped by addition of SDS sample buffer. Proteins were separated by electrophoresis on 0.1% SDS-15% polyacrylamide gels, which were subsequently dried and exposed overnight to a storage phosphor screen (STORM).

ATP-dependent NPr phosphorylation assays were performed in 50 µl reaction mixtures containing 50 mM Tris-HCl pH 7.4, 5 mM MgCl_2_, 25 µM [γ-^32^P]ATP or [^32^P]PPi and varying amounts of either FBP or potassium phosphate. The assay mixtures were incubated for 20 min at 37°C and the reaction was stopped by addition of SDS sample buffer. Proteins were separated on 0.1% SDS-15% polyacrylamide gels. After electrophoresis, gels were boiled for 10 min in 0.5 N HCl, dried and exposed overnight to a storage phosphor screen.

For P-Ser-NPr dephosphorylation assays, P-Ser-NPr was obtained by incubating *B. melitensis* (His)_6_-NPr with (His)_6_-HprK/P for 30 min at 37°C in 50 mM Tris-HCl pH 7.4, 5 mM MgCl_2_, 5 mM ATP and 25 mM FBP. HprK/P was subsequently inactivated by keeping the reaction mixture for 10 min at 65°C. P-Ser-NPr was then loaded on a PD-10 column (GE Healthcare), eluted with 20 mM NH_4_HCO_3_ to eliminate ATP, and lyophilized. Dephosphorylation assays were carried out in reaction mixtures containing 50 mM Tris-HCl pH 7.4, 5 mM MgCl_2_, 3 **µ**g of P-Ser-NPr, 0.45 µg of HprK/P and various concentrations of potassium phosphate. The assay mixtures were incubated for 30 min at 37°C before HprK/P was heat-inactivated for 10 min at 65°C. The different forms of NPr were separated by electrophoresis on non-denaturing 12.5% polyacrylamide gels, followed by staining with Coomassie Blue.

### RNA isolation and RT-PCR assays

Extraction of B. melitensis 16M total RNA was performed on cultures (40 ml) grown to late exponential growth phase in 2YT. Bacterial cells were harvested by centrifugation for 10 min at 3500 rpm, and resuspended in 100 µl 10% SDS, 20 µl proteinase K (20 mg/ml) and RNaseOUT™ (Invitrogen Life-Technologies), and incubated for 1 hour at 37°C. Total RNA was then extracted using TRIzol® reagent. Contaminating genomic DNA was digested with DNase I DNA-free (Ambion) before the enzyme was inactivated by DNase Inactivation Reagent (Ambion). Reverse transcriptions (RT) were performed as follows: random primers (200 ng/µl) (Invitrogen Life-Technologies) and dNTP mix (10 mM each dNTP) (Invitrogen Life-Technologies) were added to 3–4 µg of DNase-treated total RNA and the mixture was incubated at 65°C for 10 min. 5X First-Strand buffer, DTT (0.1 M) and RNaseOUT™ (Invitrogen Life-Technologies) were added to the solution, which was incubated at 25°C for 2 min. Finally, SuperScript™ reverse transcriptase (Invitrogen Life-Technologies) was added and incubated for 10 min at 25°C and 50 min at 42°C. The enzyme was inactivated by heating to 70°C for 15 min. To remove RNA hybridized to the cDNA, E. coli RNase H (Invitrogen Life-Technologies) was added to the RT reaction. A control reaction containing the same components but no reverse transcriptase was included to check for DNA contamination. The cDNA products (2 µl) were then used in a PCR performed in a final volume of 30 µl and containing 1.25 U of GoTaq DNA polymerase (Promega), dNTP mix (5 mM each), and 10 pmol of each primer. A PCR control in which B. melitensis 16M genomic DNA was used as template was included. The amplification consisted of one cycle of 5 min at 95°C, followed by 35 cycles of 30 sec at 95°C, 30 sec at annealing temperature (depending on the primers used), 90 sec at 72°C, and a final step of 10 min at 72°C. Primers used in this experiment are listed in Table S2.

Concerning the microarray data for the *virB* expression, RNA was reverse transcribed, labeled and hybridized by NimbleGen™ Systems, Inc using the catalogue design for *B. melitensis* 16M chromosomes I (NC_003317) and II (NC_003318) with 20 probes per gene (10 perfect matches and 10 mismatches). Each probe (24 mer) was replicated three times on a chip at a random position (design includes random GC probes). Duplicate samples of each strain were processed. Analysis of the data were performed “mutatis mutandis” as described previously [71].

### Gateway® cloning of genes of interest in Y2H vectors

For Y2H interaction tests, each protein of interest (YFP) was fused with both AD and BD domains of the transactivator Gal4. Entry vectors pDONR201 of the ORFeome [89] corrresponding to detected genes of interest (YFG) (Table S1) were subcloned in Y2H destination vectors pVV212 and pVV213 (Table S1) [91]. LR recombination procedure was performed as recommended by the manufacturer (Invitrogen Life-Technologies) to fuse YFP with both Gal4-BD (in pVV212) and Gal4-AD (in pVV213) generating plasmids pVV212-YFG and pVV213-YFG [54].

### Yeast two hybrid assay

Haploïd yeast Mav103 and Mav203 [92] were transformed with pVV212-YFG and pVV213-YFG respectively, and selected on SD-W (tryptophan omission medium) and SD-L (leucin omission medium) respectively. Mating of two plasmid-carrying yeasts was then carried out, and SD-LW (leucin and tryptophan omission medium) was then used to select diploids containing both pVV212 and pVV213. Two growth tests can be used to detect physical interactions between proteins, i.e. (i) SD-HLW + 3-AT (medium without histidine and with 20 to 50 mM triaminotriazole (3-AT) and (ii) SD-ULW (medium without uracil). The additional *lacZ* reporter gene was used to detect interactions by performing β-galactosidase coloration assays. For all Y2H assays used in this study, except for the interaction test between PTS proteins and BvrR and BvrS, β-galactosidase coloration tests were performed as follow. Diploid yeasts were plated on a nitrocellulose filter laid on a yeast peptone dextrose (YPD) plate and grown overnight at 30°C. The filter was then placed in liquid nitrogen to lyse the cells, transferred on a new plate containing two Whatman papers saturated with β-galactosidase assay solution (for each plate 5 ml of Z-buffer, 120 µl of 4% X-gal and 13 **µ**l of 100% β-mercaptoethanol), and finally incubated at 37°C. In the case of interaction tests between PTS proteins and BvrR or BvrS, β-galactosidase coloration tests were performed using an overlay plate assay as described in [93].

### Y2H screen against the ORFeome of *B. melitensis* 16M

Briefly, entry vectors pDONR201 of the ORFeome [89] were pooled by 48 (half of a 96-wells plate) to obtain 69 pools borne in a single 96-well plate. Each pool was subcloned in the Y2H vector pVV213 in order to fuse *B. melitensis* proteins to the Gal4 activating domain [91] using LR. Pools of pVV213 were used to transform the haploïd yeast Mav203. To select interacting partners of our proteins of interest, mating was performed using the pools of Mav203 containing pVV213 plasmids and Mav103 strains containing pVV212 bearing our genes of interest. Diploïds were selected using SD-LW medium. As a first screen for selecting interactions, an overnight culture of the diploïds was grown in SD-HLW medium at 30°C under shaking, and plated on SD-HLW with 20 mM 3-AT. Five diploid controls were used for this Y2H assay containing: (i) empty pVV212 and pVV213 (negative control), (ii) a weak interaction (BD-Rb and AD-E2F), (iii) a strong interaction (BD-Fos and AD-jun), (iv) complete Gal4 with empty pVV213 and (v) a strong interaction (BD-DP and AD-E2F) [94]. For each pool that showed growth, a maximum of four clones was cultivated in SD-HLW and plated on SD-LW (for a back-up), SD-HLW with 20 mM of 3-AT, on SD-ULW and on nitrocellulose filters placed on a YPD plate for β-galactosidase coloration tests. Clones that were positive for at least two Y2H tests were selected and PCR was carried out with primers iGAl4AD and Gal4term to amplify the inserts cloned in the pVV213-derived plasmids. Finally, the PCR products were sequenced using primer iGAl4AD to identify the putative interacting partner. Interactions between our proteins of interest and newly detected partners were confirmed as described in the Y2H assay.

### DivIVA interaction test

The plasmids used for the experiments were obtained as follows. The pKD46 vector was used to amplify the pBad promoter sequence with oligonucleotide pairs (Fpbad and Rpbad) (Table S2). The pBad-PCR product was cloned into pSK*ori*T*cat* digested with *Eco*RV. The pZD6 vector was used to amplify the *divIVA-gfp* fusion with oligonucleotide pairs (FdivIVA and Rgfp) (Table S2). The *divIVA-gfp* PCR product was cloned into the pGEMTeasy vector. The pGEMT-*divIVA-gfp* vector was digested with *Nhe*I and *Kpn*I and the fused genes were cloned into pSK*ori*T*cat* –pBad digested with the same enzymes. The plasmid pSK*ori*T*cat* –pBad-*divIVA-gfp* was used as positive control.

The entry vector bearing the *ptsM* gene (pDONR201- *ptsM* – Table S1) was taken from the *B. melitensis* ORFeome. This vector was used to amplify by PCR the *ptsM* gene with oligonucleotide pairs (FptsM and RptsM) (Table S2). The *ptsM*-PCR product was cloned into vector pSK*ori*T*cat* digested with *Eco*RV, giving plasmid pSK*ori*T*cat*–*ptsM* encoding the EIIA^Man^-like protein. The pZD6 vector was used to amplify the *gfp* gene with oligonucleotide pairs (Fgfp and Rgfp) (Table S2). The *gfp*-PCR product was cloned into the pGEMTeasy vector. The pGEMT-*gfp* vector was digested with *Bgl*II and *Kpn*I to get *gfp* which was cloned into pSK*ori*T*cat* – *ptsM* digested with the same enzymes. The pSK*ori*T*cat* –*ptsM-gfp* vector was digested with *Hin*dIII and *Kpn*I and cloned into pMR10*kan* digested with the same enzymes.

The pZD6 vector was used to amplify the *divIVA* gene with oligonucleotide pairs FdivIVA and RdivIVA (Table S2). The *divIVA*-PCR product was cloned into pSK*ori*T*amp* digested with *EcoRV*. The pSK*ori*T*amp*-*divIVA* vector was digested with *Nhe*I and *Hin*dIII and *divIVA* was cloned into pSK*ori*T*cat*-pBad digested with the same enzymes.

*B. melitensis* genomic DNA was used to amplify by PCR the *sucA* gene with oligonucleotide pairs (FsucA and RsucA) (Table S2). The *sucA*-PCR product was cloned into the pGEM11Zf vector. The pGEM11Zf-*sucA* vector was digested with *Hin*dIII and *Xho*I and cloned into pSK*ori*T*cat* –pBad-*divIVA* digested with the same enzymes. The correct sequence of all PCR products was confirmed by DNA sequencing.

The two plasmids, pSK*ori*T*cat* –pBad-*divIVA-sucA* and pMR10*kan-ptsM-gfp*, were used to co-transform *E. coli* DH10B competent cells. The resulting strain was cultivated in 10 ml SOB medium (tryptone 2%, yeast extract 0.5%, NaCl 0.058%, KCl 0.019% and MgCl_2_ 0.19%) with chloramphenicol (20 µg/ml) until the OD_600_ reached 0.1. Arabinose (10 mM) induction was performed during three hours before the microscopic observation.

### Rabbit immunization

In order to produce monospecific polyclonal antisera against EIIA^Ntr^, rabbits were immunized with the purified protein (50 µg per dose), initially in the presence of complete Freund's adjuvant and on days 30 and 60 with incomplete Freund's adjuvant. Rabbits were bled 1 week after the last injection.

### Detection of *in vivo* phosphorylated EIIA^Ntr^

Wild-type strain and Δ*hprK*, Δ*ptsP* and Δ*ptsO* mutants were cultivated in 100 ml 2YT until reaching an OD_600_ of 0.7-0.8. Cells were harvested by centrifugation, washed and disrupted by vortexing with glass beads. Cell debris was removed by centrifugation and the supernatants were used for the phosphorylation tests. When carrying out phosphorylation experiments with purified His-tagged EIIA^Ntr^ we had previously observed that EIIA^Ntr^ phosphorylated with PEP, EI and NPr migrates significantly faster on non-denaturing polyacrylamide gels than unphosphorylated EIIA^Ntr^. Aliquots of the crude extracts containing 60 µg of protein were therefore loaded on a non-denaturing polyacrylamide gel and separated by electrophoresis. Proteins were transferred onto a nitrocellulose membrane, which was processed for immunodetection with a polyclonal antibody against EIIA^Ntr^ and a secondary antibody coupled to horseradish peroxidase before carrying out ECL revelation (GE Healthcare).

### Construction of Δ*pts* mutants and complementation strains

*B. melitensis* 16M *pts* knock out mutants were obtained by gene replacement as previously described [46]. For each *pts* gene, upstream and downstream regions (about 500 bp) flanking the gene were PCR amplified from *B. melitensis* 16M genomic DNA by using appropriate primers (Table S2). A second PCR was used to associate the two PCR products by cohesive ends. The final PCR product that carries a *Bgl*II site between the upstream and the downstream regions was inserted into the *Not*I site of pSK*oriTcat* (Table S1). The *aphA4* cassette [46] was excised from pUC4*aphA4* (Table S1) with *Bam*HI and subsequently cloned into the *Bgl*II site to generate plasmid pSK*oriTcat*-Δ*pts* (or -Δ*hprK*) (Table S1). These constructs were used to transform *E. coli* strain S17-1 and subsequently introduced into *B. melitensis* 16M by mating. Clones exhibiting a double recombination phenotype (Cm^s^ Kan^r^) were selected and their genotypes were verified by PCR and by Southern blot analysis using appropriate probes. The complementation plasmids pRH001-*ptsP* and -*ptsO* (Table S1) were constructed by using the Gateway™ technique (Invitrogen Life-Technologies). LR recombination cloning was carried out as recommended by the manufacturer (Invitrogen Life-Technologies) in order to insert selected genes in pRH001 using pDONR201-*ptsP* and -*ptsO*, -*ptsO*_H30A_ and -*ptsO*_S61A_ as entry vectors (Table S1). The resulting vectors pRH001-*ptsP* and *-ptsO,* -*ptsO*_H30A_ and -*ptsO*_S61A_ (Table S1) were transferred by mating into the Δ*ptsP* or Δ*ptsO* mutants to generate the complemented strains Δ*ptsP/ptsP*, Δ*ptsO/ptsO*, Δ*ptsO//ptsO*_H30A_ and Δ*ptsO/ptsO*_S61A_. In parallel, pMR10*cat* (Table S1) was transfered into *B. melitensis* 16M wild-type, Δ*ptsP* and Δ*ptsO* strains by mating.

### Detection of VirB5 and VirB10 proteins by Western blot analyses

For VirB detection in total lysates of *B. melitensis* 16M and various mutants, strains were grown overnight at 37°C in 2YT complex medium and then diluted and grown at 37°C until late log phase (OD_600_ 0.8–1.2). Aliquots of the cultures were kept for 1 hour at 80°C in order to inactivate cell functions and then adjusted to the same OD_600_. Following SDS-polyacrylamide gel electrophoresis and Western blot analysis, immunodetection of VirB5 and VirB10 in total lysates was performed with rabbit polyclonal anti-VirB5 and -VirB10 antisera [53] at respective dilutions of 1/5000 and 1/2000. Immunodetection with a monoclonal antibody anti-Omp 89 [95] was used as loading control.

## Supporting Information

Figure S1 Multiple sequence alignment of N-terminal portion of enzyme I^Ntr^. The predicted PEP-dependent phosphorylated histidine of enzymes I^Ntr^ regarding multiple alignment with paralogous enzymes I is marked by an asterisk and shaded, and the conserved region surrounding it is boxed. The predicted N-terminal GAF domain homologous to the NifA-sensory domain of *Azotobacter vinelandii* is underlined and limited by two vertical bars. Red residues are identical for the five proteins, whereas green and blue residues are strongly or weakly similar, respectively. (EInSme), *Sinorhizobium meliloti*, (EInAtu) *Agrobacterium tumefaciens*, (EInBme) *Brucella melitensis* and (EInEco) *Escherichia coli*.
(0.86 MB TIF)

Figure S2 Multiple sequence alignment of enzyme IIA^Ntr^. Conserved histidine predicted to be phosphorylated by NPr in *E. coli*, *S. meliloti*, *A. tumefaciens* and *B. melitensis* is marked by an asterisk and shaded. The well-conserved region surrounding the putative phosphorylation site is boxed. Red residues are identical for the five proteins, whereas green and blue residues are strongly or weakly similar, respectively. (EInSme), *Sinorhizobium meliloti*, (EInAtu) *Agrobacterium tumefaciens*, (EInBme) *Brucella melitensis* and (EInEco) *Escherichia coli*.
(0.43 MB TIF)

Figure S3 Mutiple sequence alignment of enzyme IIA^Man^. Conserved histidine phosphorylated by HPr in *E. coli* that is predicted to be phosphorylated by NPr in *S. meliloti, A. tumefaciens* and *B. melitensis* is marked by an asterisk and shaded. Red residues are identical for the five proteins, whereas green and blue residues are strongly or weakly similar, respectively. *Sinorhizobium meliloti* (IIAmSme), *Agrobacterium tumefaciens* (IIAmAtu), *Brucella melitensis* (IIAmBme) and domain IIA of enzyme IIABMan from *Escherichia coli* (IIAmEco).
(0.38 MB TIF)

Figure S4 Multiple sequence alignment of NPr proteins. The conserved histidine residue phosphorylated by enzyme I on HPr from *B. subtilis* and *E. coli*, that is predicted to be phosphorylated by enzyme I^Ntr^ on NPr proteins from *E. coli, S. meliloti, A. tumefaciens* and *B. melitensis* is marked by an asterisk and shaded. Similarly, the conserved serine residue phosphorylated by HprK/P on HPr protein from *B. subtilis*, that is predicted to be phosphorylated by HprK/P on NPr proteins from *S. meliloti, A. tumefaciens* and *B. melitensis* is marked by an asterisk and shaded. The consensus sequences surrounding these two predicted phosphorylation sites are boxed. Red residues are identical for the five proteins, whereas green and blue residues are strongly or weakly similar, respectively. *Sinorhizobium meliloti* (NPrSme), *Agrobacterium tumefaciens* (NPrAtu), *Brucella melitensis* (NPrBme), *Escherichia coli* (NPrEco) and HPr proteins from *E. coli* (HPrEco) and *Bacillus subtilis* (HPrBsu).
(0.42 MB TIF)

Figure S5 Multiple sequence alignment of HprK/P proteins. The conserved Walker A motif which binds ATP, PPi and Pi in HprK/P proteins is boxed (155-GDSGVGGKS-162 in *L. casei HprK/P*)). The HprK/P signature sequence, whose consensus is (I,L,M)E(I,V)RG(I,L,M,V)G(I,V)(I,L,M) (residues 203 to 211 in *L. casei HprK/P*), is also boxed. An additional conserved region present in HprK from Gram positive bacteria and playing an important role in phosphorylase activity of the protein is underlined. This region is not conserved in HprK/P from α-proteobacteria. Shaded residues are amino acids that were shown to be required either for kinase or phosphorylase activities. Red residues are identical for the five proteins, whereas green and blue residues are strongly or weakly similar, respectively. *Sinorhizobium meliloti* (HprKSme), *Agrobacterium tumefaciens* (HprKAtu), *Brucella melitensis* (HprKBme) and C-terminal portion of HprK/P proteins from *Lactobacillus casei* (HprKLca) and *Bacillus subtilis* (HprKBsu).
(0.58 MB TIF)

Figure S6 Interaction matrix for PTS proteins, HprK/P and the two-component system BvrS/BvrR. AD-P =  protein of interest fused with the activating domain (AD) of Gal4; BD-P =  protein of interest fused with the DNA binding domain (BD) of Gal4. Interactions demonstrated with one or two reporter genes (*lacZ* or *HIS3*) are shown in grey and black respectively.
(0.04 MB DOC)

Table S1 Strains and plasmids used in this study.
(0.10 MB DOC)

Table S2 List of the primers used in this study.
(0.11 MB DOC)
