# Supplementary figures and images for: Functional Characterization of the Incomplete Phosphotransferase System (PTS) of the Intracellular Pathogen Brucella melitensis
Source: PLoS One. 2010 Sep 10;5(9):e12679. doi: 10.1371/journal.pone.0012679 (PMC2937029; doi:10.1371/journal.pone.0012679)

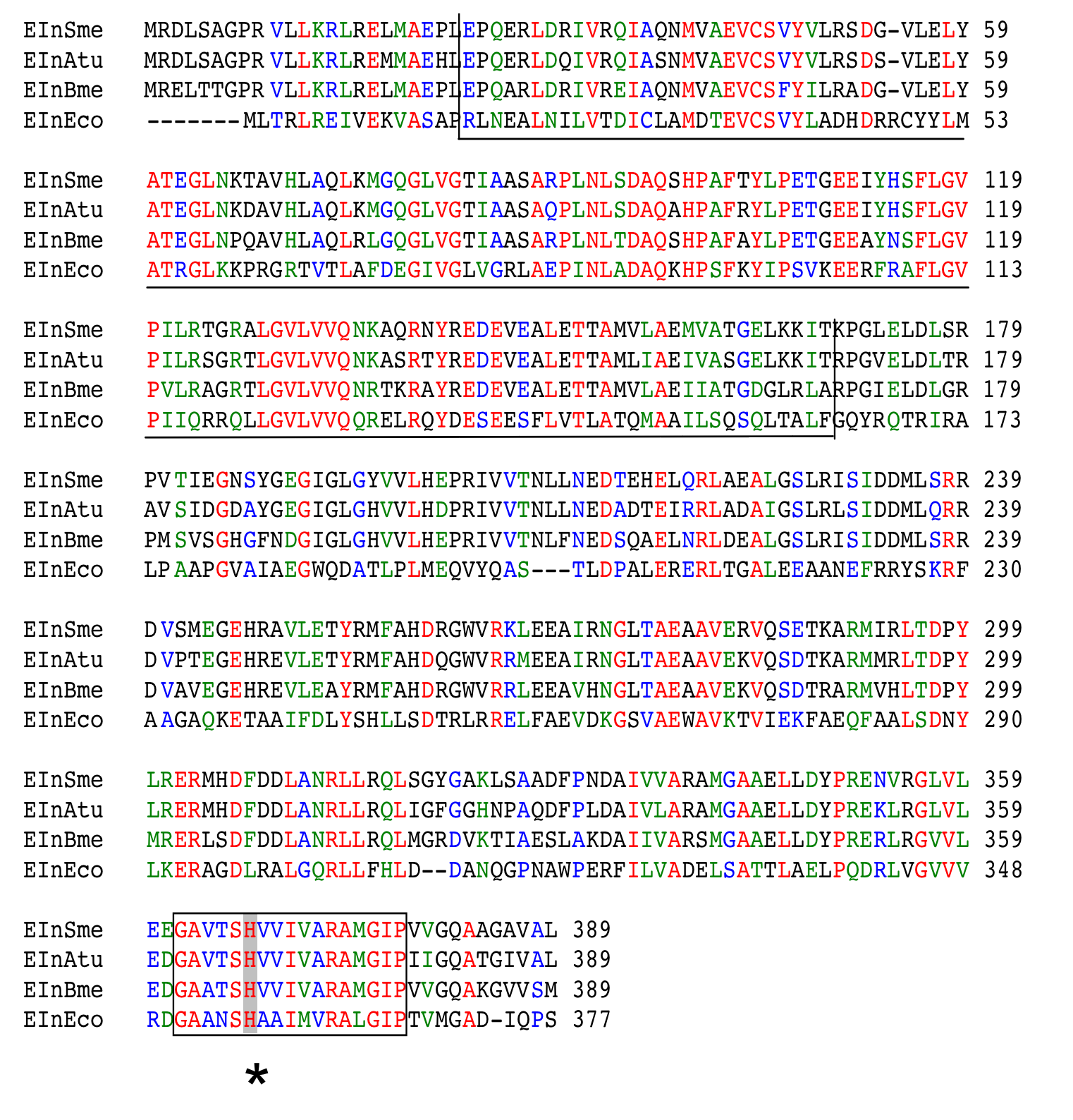

Supplement: Figure S1 — Multiple sequence alignment of N-terminal portion of enzyme INtr. The predicted PEP-dependent phosphorylated histidine of enzymes INtr regarding multiple alignment with paralogous enzymes I is marked by an asterisk and shaded, and the conserved region surrounding it is boxed. The predicted N-terminal GAF domain homologous to the NifA-sensory domain of Azotobacter vinelandii is underlined and limited by two vertical bars. Red residues are identical for the five proteins, whereas green and blue residues are strongly or weakly similar, respectively. (EInSme), Sinorhizobium meliloti, (EInAtu) Agrobacterium tumefaciens, (EInBme) Brucella melitensis and (EInEco) Escherichia coli. (0.86 MB TIF) [file pone.0012679.s001.tif]

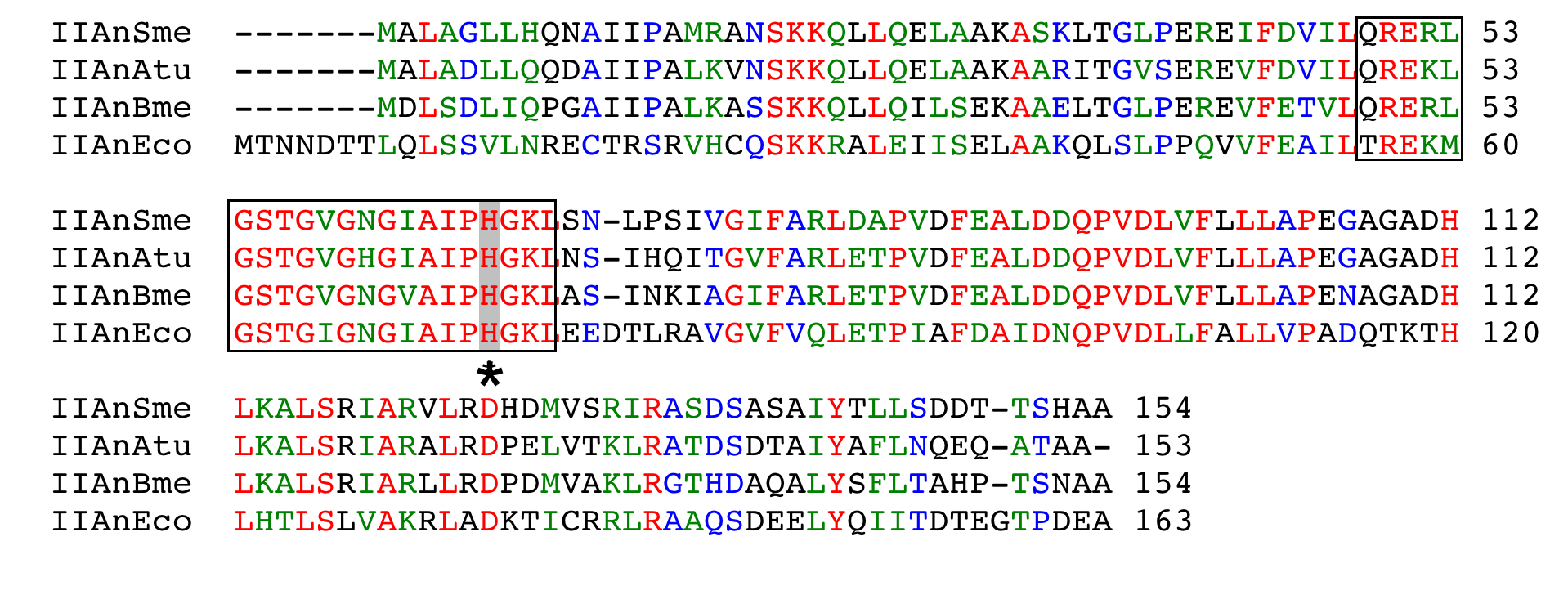

Supplement: Figure S2 — Multiple sequence alignment of enzyme IIANtr. Conserved histidine predicted to be phosphorylated by NPr in E. coli, S. meliloti, A. tumefaciens and B. melitensis is marked by an asterisk and shaded. The well-conserved region surrounding the putative phosphorylation site is boxed. Red residues are identical for the five proteins, whereas green and blue residues are strongly or weakly similar, respectively. (EInSme), Sinorhizobium meliloti, (EInAtu) Agrobacterium tumefaciens, (EInBme) Brucella melitensis and (EInEco) Escherichia coli. (0.43 MB TIF) [file pone.0012679.s002.tif]

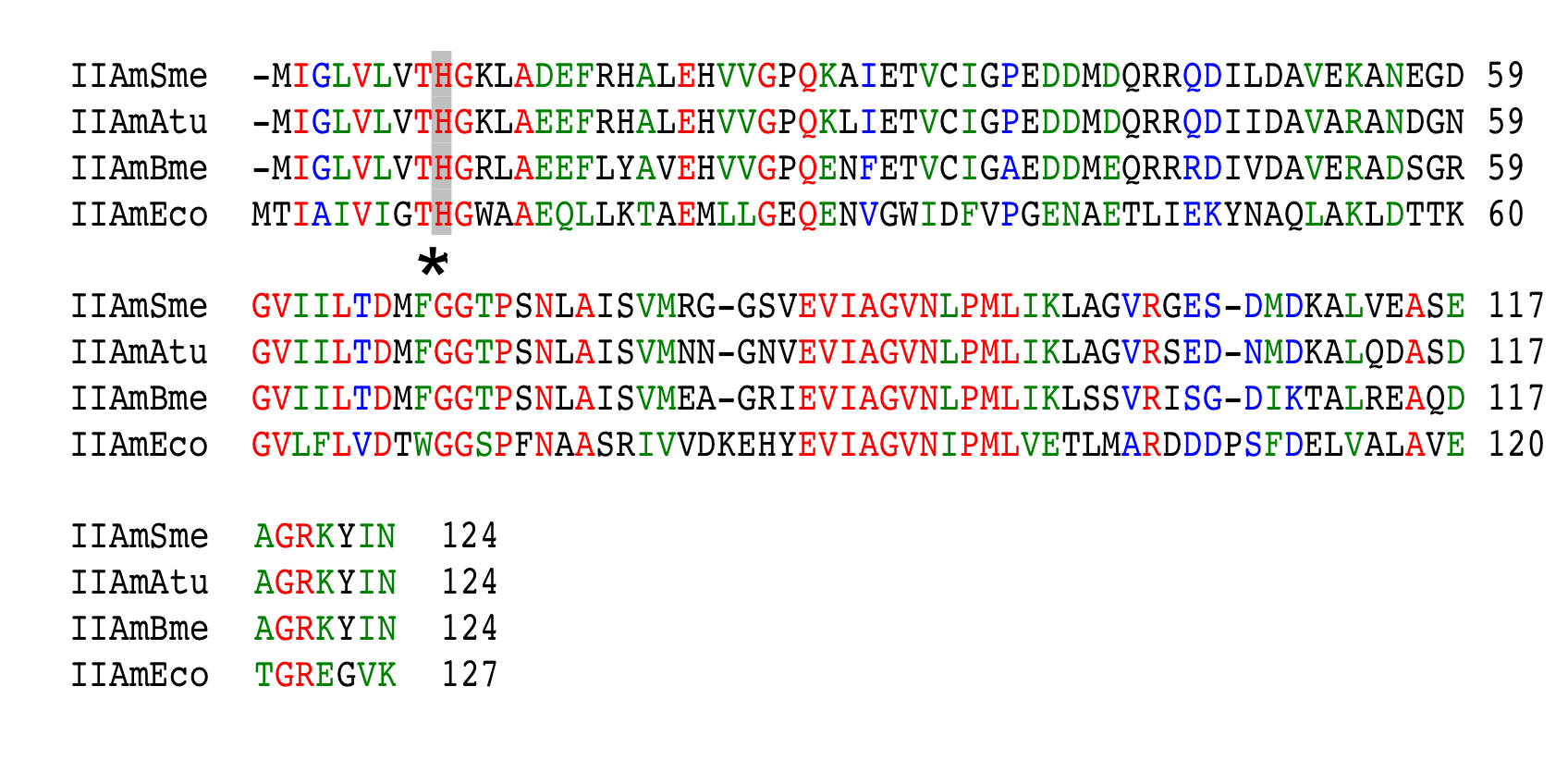

Supplement: Figure S3 — Mutiple sequence alignment of enzyme IIAMan. Conserved histidine phosphorylated by HPr in E. coli that is predicted to be phosphorylated by NPr in S. meliloti, A. tumefaciens and B. melitensis is marked by an asterisk and shaded. Red residues are identical for the five proteins, whereas green and blue residues are strongly or weakly similar, respectively. Sinorhizobium meliloti (IIAmSme), Agrobacterium tumefaciens (IIAmAtu), Brucella melitensis (IIAmBme) and domain IIA of enzyme IIABMan from Escherichia coli (IIAmEco). (0.38 MB TIF) [file pone.0012679.s003.tif]

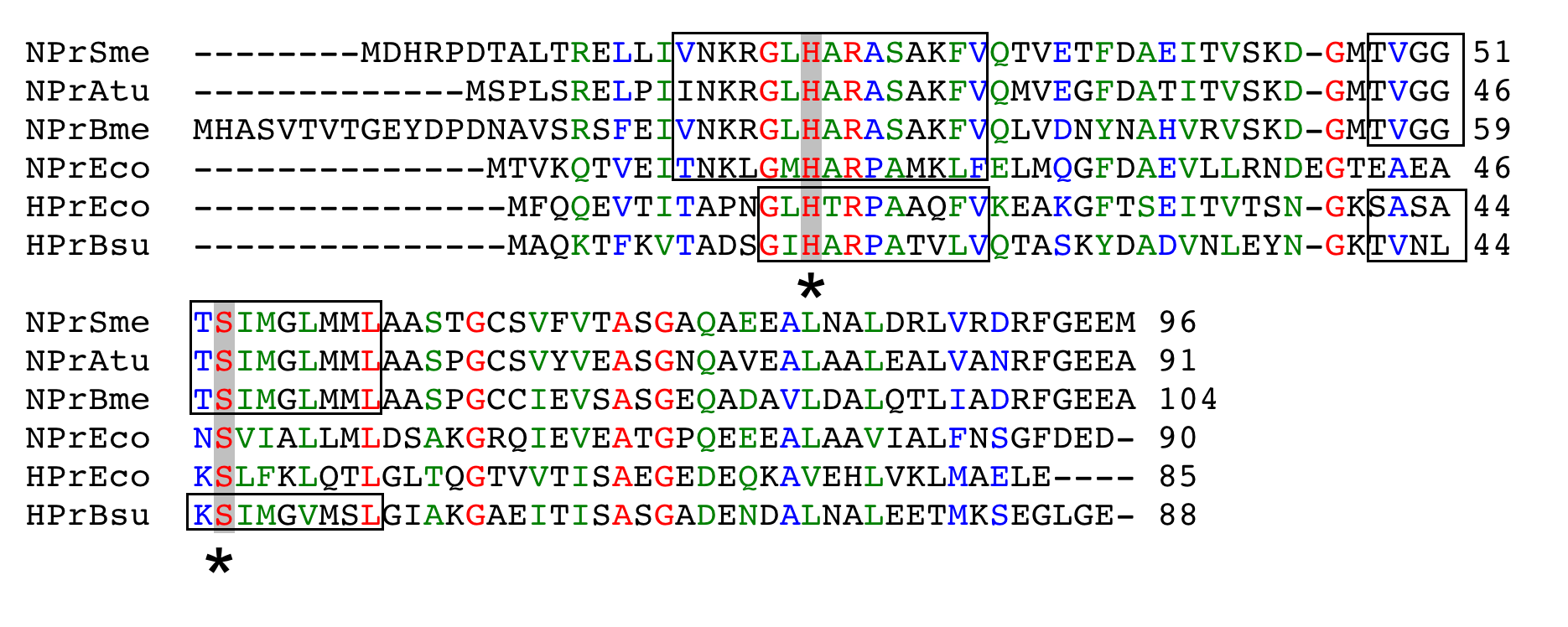

Supplement: Figure S4 — Multiple sequence alignment of NPr proteins. The conserved histidine residue phosphorylated by enzyme I on HPr from B. subtilis and E. coli, that is predicted to be phosphorylated by enzyme INtr on NPr proteins from E. coli, S. meliloti, A. tumefaciens and B. melitensis is marked by an asterisk and shaded. Similarly, the conserved serine residue phosphorylated by HprK/P on HPr protein from B. subtilis, that is predicted to be phosphorylated by HprK/P on NPr proteins from S. meliloti, A. tumefaciens and B. melitensis is marked by an asterisk and shaded. The consensus sequences surrounding these two predicted phosphorylation sites are boxed. Red residues are identical for the five proteins, whereas green and blue residues are strongly or weakly similar, respectively. Sinorhizobium meliloti (NPrSme), Agrobacterium tumefaciens (NPrAtu), Brucella melitensis (NPrBme), Escherichia coli (NPrEco) and HPr proteins from E. coli (HPrEco) and Bacillus subtilis (HPrBsu). (0.42 MB TIF) [file pone.0012679.s004.tif]

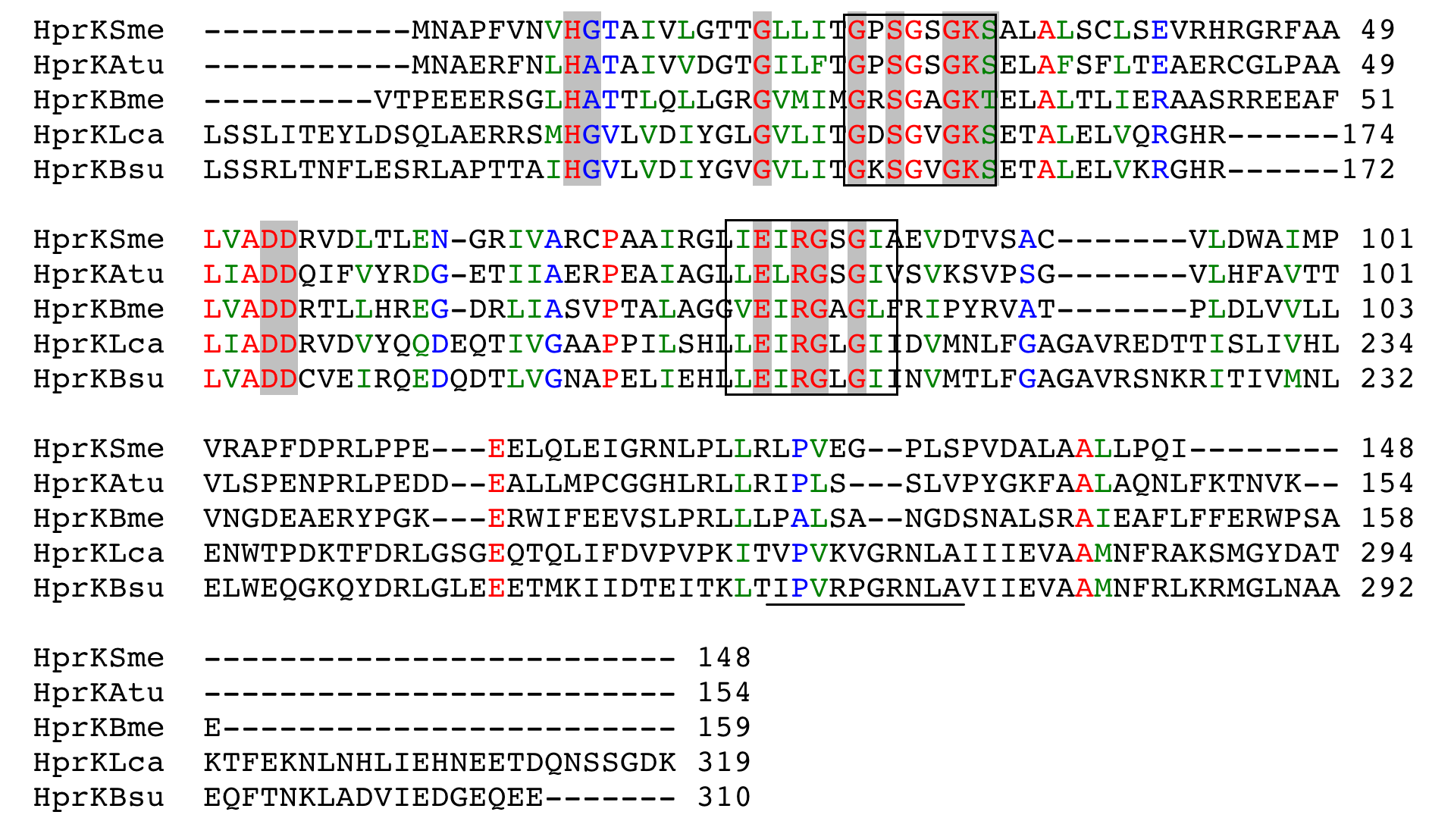

Supplement: Figure S5 — Multiple sequence alignment of HprK/P proteins. The conserved Walker A motif which binds ATP, PPi and Pi in HprK/P proteins is boxed (155-GDSGVGGKS-162 in L. casei HprK/P)). The HprK/P signature sequence, whose consensus is (I,L,M)E(I,V)RG(I,L,M,V)G(I,V)(I,L,M) (residues 203 to 211 in L. casei HprK/P), is also boxed. An additional conserved region present in HprK from Gram positive bacteria and playing an important role in phosphorylase activity of the protein is underlined. This region is not conserved in HprK/P from α-proteobacteria. Shaded residues are amino acids that were shown to be required either for kinase or phosphorylase activities. Red residues are identical for the five proteins, whereas green and blue residues are strongly or weakly similar, respectively. Sinorhizobium meliloti (HprKSme), Agrobacterium tumefaciens (HprKAtu), Brucella melitensis (HprKBme) and C-terminal portion of HprK/P proteins from Lactobacillus casei (HprKLca) and Bacillus subtilis (HprKBsu). (0.58 MB TIF) [file pone.0012679.s005.tif]
