# Supplementary material for: Functional Characterization of the Incomplete Phosphotransferase System (PTS) of the Intracellular Pathogen Brucella melitensis
Source: PLoS One. 2010 Sep 10;5(9):e12679. doi: 10.1371/journal.pone.0012679 (PMC2937029; doi:10.1371/journal.pone.0012679)
Supplement: Figure S6 — Interaction matrix for PTS proteins, HprK/P and the two-component system BvrS/BvrR. AD-P = protein of interest fused with the activating domain (AD) of Gal4; BD-P = protein of interest fused with the DNA binding domain (BD) of Gal4. Interactions demonstrated with one or two reporter genes (lacZ or HIS3) are shown in grey and black respectively. (0.04 MB DOC) [file pone.0012679.s006.doc]

#

Fig 6S: Interaction matrix for PTS proteins, HprK/P and the two-component system BvrS/BvrR. AD-P =protein of interest fused with the activating domain (AD) of Gal4; BD-P= protein of interest fused with the DNA binding domain (BD) of Gal4. Interactions demonstrated with one or two reporter genes (*lacZ* or *HIS3*) are shown in grey and black respectively.
