# Supplementary material for: Functional Characterization of the Incomplete Phosphotransferase System (PTS) of the Intracellular Pathogen Brucella melitensis
Source: PLoS One. 2010 Sep 10;5(9):e12679. doi: 10.1371/journal.pone.0012679 (PMC2937029; doi:10.1371/journal.pone.0012679)
Supplement: Table S1 — Strains and plasmids used in this study. (0.10 MB DOC) [file pone.0012679.s007.doc]

**TABLE S1: Strains and plasmids used in this study**

| **Strains/plasmids** | **Descriptions** | **Reference/source** |
| --- | --- | --- |
| **Strains** |  |  |
| ***Brucella melitensis*** |  |  |
| 16M biovar 1 | wild-type*, Nal^R^* | A. Macmillan, CVL, Weybridge, UK |
| *DptsP* | *ptsP* non-polar mutant, *Nal^R^, Kan^R^* | this study |
| *DptsO* | *ptsO* non-polar mutant, *Nal^R^, Kan^R^* | this study |
| *DptsN* | *ptsN* non-polar mutant, *Nal^R^, Kan^R^* | this study |
| *DhprK* | *hprK* non-polar mutant, *Nal^R^, Kan^R^* | this study |
| ***Escherichia coli*** |  | |
| DH10B | *F - mcrA, D(mrr-hsdRMS-mcrBC ), f 80dlacZDM15, DlacX74, deoR, recA1 endA1, araD139, D (ara, leu )7697, galU, galK, l - , rspL, nupG (Sm ^R^ )* | Invitrogen Life-Technologies |
| DB3.1 | *F - gyrA 462 endA -D(sr 1-recA ) mcr B mrr hsd 20(r B,m B-) sup E44 ara 14gal K2 lac Y1 pro A2 rps L 1-leu mtl 1* | Invitrogen Life-Technologies |
| S17-1 | *l pir [recA thi pro hsd R- M+ RP4::2-Tc::Mu::Km Tn7 lysogenized with l pir phage] (Sm ^R^ )* | Simon *et al*., 1983 |
| NM522 | *supE thi-1 D (lac-proAB) D (merB-hsdSM)5(r K^_^ m _K_^+^ ) [F’proABlacI ^q^ ZDM15*] | Stratagene |
| **Yeast** |  | |
| **(*S. cerevisiae*)** |  | |
| *Mav103* | (*Mata*) : *leu2-3, 112 trp-901 his3∆200 ade2-1 gal4∆ gal80∆ SPAL10::URA3 GAL1::lacZ GAL1::HIS3-@LYS2 can1R cyh2R* | Walhout and Vidal, 2001 |
| *Mav203* | *(Mat* α*) : leu2-3, 112 trp-901 his3∆200 ade2-1 gal4∆ gal80∆ SPAL10::URA3 GAL1::lacZ GAL1::HIS3-@LYS2 can1R cyh2R* | Walhout and Vidal, 2001 |
| **Plasmids** |  |  |
| pDONR201 | plasmid bearing toxic cassette *ccdB* flanked by *attP1* and *attP2* recom-bination sites for Gateway_TM_ cloning, and kanamycine resistance marker | Invitrogen |
| pDONR201-*ptsP* | pDONR201-derived bearing *ptsP* wt allele | This study |
| pDONR201-*ptsO* | pDONR201-derived bearing ptsO wt allele | This study |
| pDONR201-*ptsO* H30A | pDONR201-derived bearing *ptsO* H30A allele | This study |
| pDONR201-*ptsO* S61A | pDONR201-derived bearing *ptsO* S61A allele | This study |
| pDONR201-*ptsN* | pDONR201-derived bearing *ptsN* wt allele | Dricot *et al*.,2004 |
| pDONR201-*ptsM* | pDONR201-derived bearing *ptsM* wt allele | Dricot *et al*.,2004 |
| pDONR201-*hprK* | pDONR201-derived bearing *hprK* wt allele | Dricot *et al*.,2004 |
| pQE30 | overexpression vector bearing ampicillin resistance marker | Quiagen |
| pSP50 | pQE30-derived bearing *ptsO* wt allele | This study |
| pSP50H30A | pQE30-derived bearing *ptsO* H30A allele | This study |
| pSP50S61A | pQE30-derived bearing *ptsO* S61A allele | This study |
| pSP51 | pQE30-derived bearing *ptsN* | This study |
| pSP52 | pQE30-derived bearing *ptsM* | This study |
| pSP53 | pQE30-derived bearing *hprK* | This study |
| pUC4*aphA4* | pUC4K-derived (Pharmacia) bearing non-polar cassette *aphA4* | Dozot *et al.,* 2006 |
| pSK*ori* T*cat* | pBluescript SK(-)-derived, bearing RP4 conjugative transfer origin oriT and chloramphenicol resistance marker cat | Danese and Lestrate, unpublished |
| pSK*ori* T*cat* -* Δ ptsP* | pSK*ori* T*cat* -derived bearing *Δ ptsP* ::*aphA4* | This study |
| pSK*ori* T*cat* -* Δ ptsO* | pSK*ori* T*cat* -derived bearing *Δ ptsO* ::*aphA4* | This study |
| pSK*ori* T*cat* -* Δ ptsN* | pSK*ori* T*cat* -derived bearing *Δ ptsN* ::*aphA4* | This study |
| pSK*ori* T*cat* -* Δ hprK* | pSK*ori* T*cat* -derived bearing *D hprK* ::*aphA4* | This study |
| pMR10-*cat* | pMR10-derived bearing chloramphenicol resistance marker *cat* | R. Roberts, unpublished |
| pRH001 | pMR10*-cat* derived bearing toxic cassete *ccdB* flanked by *attR1* and *attR2* recombination sites for GatewayTM cloning (Invitrogen Life-Technologies) | Hallez *et al*., 2007 |
| pRH001-*ptsP* | pRH001-derived bearing *ptsP* allele | This study |
| pRH001-*ptsO* | pRH001-derived bearing *pts0* allele | This study |
| pGEMTeasy |  | Promega Inc. |
| pGEM11Zf |  | Promega Inc. |
| pSK*ori*T*kan* | pBluescript SK(-)-derived, bearing RP4 conjugative transfer origin oriT and kanamycine resistance marker *kan* | Haine, 2005 |
| pMR10-*kan* | pMR10-derived bearing kanamycine resistance marker *kan* | R. Roberts, unpublished |
| pZD6 | Crbr Kanr, pMMB22 (IncQ) with Ptac-divIVA::GFP | Ding *et al.*, 2002 |
| pKD46 | Red recombinase plasmid pKD46 | Datsenko and Wanner, 2000 |
| pSK*ori*T*cat*-pBad-*divIVA-gfp* | pSK*ori* T*cat* -derived bearing *the fusion* DivIVA-GFP under the pBad promoter control | This study |
| pSK*ori*T*cat*-pBad-*divIVA-sucA* | pSK*ori* T*cat* -derived bearing *the fusion* DivIVA-SucA under the pBad | This study |
