# Supplementary material for: Functional Characterization of the Incomplete Phosphotransferase System (PTS) of the Intracellular Pathogen Brucella melitensis
Source: PLoS One. 2010 Sep 10;5(9):e12679. doi: 10.1371/journal.pone.0012679 (PMC2937029; doi:10.1371/journal.pone.0012679)
Supplement: Table S2 — List of the primers used in this study. (0.11 MB DOC) [file pone.0012679.s008.doc]

**Table S2:** List of the primers used in this study

| **Primers** | **Used to** | **Sequence** |
| --- | --- | --- |
| CPPCKR | amplify internal fragment (if) *pckA* | 5'-AGACCGGCATCCACAATA-3' |
| CPPCKF | amplify if *pckA* and jonction (j) *pckA-bvrR* | 5'-CGGACGGATCAGCAGATT-3' |
| OBVRR1 | amplify if *bvrR* and j *bvrR-bvrS* | 5'-TCTGGTCGAGCGTGTCAA-3' |
| CBVRR1 | amplify if *bvrR* and j *pckA-bvrS* | 5'-CGCTTCCCGGAAACGATA-3' |
| CBVRS1 | amplify if *bvrS* | 5'-GTCGCAGAGACCCAGAAA-3' |
| OBVRS1 | amplify if *bvrS* and j *bvrR-bvrS* | 5'-GCTGGACGGGAAGCTTGT-3' |
| OBVRS2 | amplify j *bvrS-hprK* | 5'-CGACCGCGTGGATATGAA-3' |
| CHPRK2 | amplify if *hprK* and j *hprK-ptsM* | 5'-GTGACGCCCGAGGAGGAA-3' |
| OHPRK2 | amplify if *hprK* and j *bvrS-hprK* | 5'-GCGGAAGGCTGACCTCTT-3' |
| ONPR4 | amplify if *ptsM* and j *ptsM-ptsO* | 5'-GATCGGACTCGTGCTTGT-3' |
| CEIIM4 | amplify if *ptsM* and j *hprK-ptsM* | 5'-TTTTCCTGTCAGGACCTGG-3' |
| CNPR4 | amplify if *ptsO* | 5'-GACCGTCACCGGCGAATA-3' |
| OEIIM4 | amplify if *ptsO* and j *ptsM-ptsO* | 5'-CGAACCTGTCGGCGATAA-3' |
| CADENF | amplify if *sahH* | 5'-TGGCTGCCCGCGAGGAAT-3' |
| CADENR | amplify if *sahH* | 5'-TTCCTCGCTCTGCGGATT-3' |
| OEIIM5 | amplify j *ptsO*-*sahH* | 5'-GTCCATCATGGGCTTGAT-3' |
| OADEN5 | amplify j *ptsO*-*sahH* | 5'-GCCATCAGGCCCGGCATT-3' |
| CEINTF | amplify if *ptsP* | 5'-ACAACCGGTCCCCGTGTA-3' |
| CEINTR | amplify if *ptsP* | 5'-CACGACAAGAACGCCTAA-3' |
| CEIINT | amplify if *ptsN* | 5'-GATCTTAGCGATCTGATT-3' |
| OEIINT | amplify if *ptsN* | 5'-TCGGGGTCGCGCAGCAAA-3' |
| GWnprF | construct pDONR201-*ptsO* | 5'-ATGCATGCGAGTGTGACC-3' |
| GWnprR | construct pDONR201-*ptsO* | 5'-GGGGACAAGTTTGTACAAAAAAGCAGGCTCGATGCATGCGAGTGTGACC-3' |
| H30AF | construct pDONR201-*ptsO* H30A | 5'-CAACAAGCGGGGGCTTGCAGCTCGCGCTTCGGC-3' |
| H30AR | construct pDONR201-*ptsO* H30A | 5'-GCCGAAGCGCGAGCTGCAAGCCCCCGCTTGTTG-3' |
| S61AF | construct pDONR201-*ptsO* S61A | 5'-GACGGTGGGCGGCACGGCCATCATGGGCTTGATG-3' |
| S61AR | construct pDONR201-*ptsO* S61A | 5'-CATCAAGCCCATGATGGCCGTGCCGCCCACCGTC-3' |
| SP65 | construct pQE30-*ptsO*, -*ptsO* H30A,  -*ptsO* S61A | 5'-GGGGGGATCCATGCATGCGAGTGTGACCGT-3' |
| SP66 | construct pQE30-*ptsO*, -*ptsO* H30A,  -*ptsO* S61A | 5'-CCCCGGTACCTCAGGCTTCCTCGCCGAA-3' |
| SP67 | construct pQE30-*ptsN* | 5'-GGGGGGATCCATGGATCTTAGCGATCTGATTC-3' |
| SP68 | construct pQE30-*ptsN* | 5'-CCCCGGTACCTCAGGCGGCGTTGGACGT-3' |
| SP69 | construct pQE30-*ptsM* | 5'-GGGGGGTCCATGATCGGACTCGTGCTTGTTACGCACGGAAGGCTGGC-3' |
| SP70 | construct pQE30-*ptsM* | 5'-CCCCGGTACCCTATTTTCCTGTCAGGACCTGGCTTGCAACATTGATA-3' |
| SP74 | construct pQE30-*hprK* | 5'-GGGGGGATCCGTGACGCCCGAGGAGGAAAG-3' |
| SP75 | construct pQE30-*hprK* | 5'-CCCCGGTACCTCACTCTGCGGAGGGCCAGC-3' |
| FPptsEI | construct pSK*oriTcat*-D*ptsP* | 5'-CCATCACCACGTCGGAAA-3' |
| RPptsEI | construct pSK*oriTcat*-D*ptsP* | 5'-AATTCCCCCGGGGAGATCTCCGTTGTCAGCTCACGCATT-3' |
| FTptsEI | construct pSK*oriTcat*-D*ptsP* | 5'-GGAGATCTCCCCGGGGGAATTGCGCATTCCTTGCGCGAA-3' |
| RTptsEI | construct pSK*oriTcat*-D*ptsP* | 5'-GCACCGCGATGCGCTTTT-3' |
| FPnpr | construct pSK*oriTcat*-D*ptsO* | 5'-GCACGCGACGGGAGCTTT-3' |
| RPnpr | construct pSK*oriTcat*-D*ptsO* | 5'-AATTCCCCCGGGGAGATCTCCGTCACACTCGCATGCATA-3' |
| FTnpr | construct pSK*oriTcat*-D*ptsO* | 5'-GGAGATCTCCCCGGGGGAATTGACAGGTTCGGCGAGGAA-3' |
| RTnpr | construct pSK*oriTcat*-D*ptsO* | 5'-CCAATCGGCCAGGCTAAT-3' |
| FPntrEIIA | construct pSK*oriTcat*-D*ptsN* | 5'-GACCCGGCTTCGCCGTTA-3' |
| RPntrEIIA | construct pSK*oriTcat*-D*ptsN* | 5'-AATTCCCCCGGGGAGATCTCCGCTGAATCAGATCGCTAA-3' |
| FTntrEIIA | construct pSK*oriTcat*-D*ptsN* | 5'-GGAGATCTCCCCGGGGGAATTGCGCAGGCCCTCTATTCA-3' |
| RTntrEIIA | construct pSK*oriTcat*-D*ptsN* | 5'-GGACAATGAAGCTCTGAA-3' |
| FPnprK | construct pSK*oriTcat*-D*HprK* | 5'-CGAAGTGCGGCGCAACAA-3' |
| RPnprK | construct pSK*oriTcat*-D*HprK* | 5'-AATTCCCCCGGGGAGATCTCCGCGTCACAGGTTCAGATA-3' |
| FTnprK | construct pSK*oriTcat*-D*HprK* | 5'-GGAGATCTCCCCGGGGGAATTCGCGCCATAGAAGCTTTT-3' |
| RTnprK | construct pSK*oriTcat*-D*HprK* | 5'-TCACGCCCCTGCCGCTAT-3' |
| RdivIVA | construct pSK*oriTcat*-pBad-*divIVA-sucA* | 5'-AAGCTTTTCCTTTTCCTCAAATACAGCGTCGA-3' |
| FdiviVA | construct pSK*oriTcat*-pBad-*divIVA-gfp*  and pSK*oriTcat-ptsM-gfp* | 5'-GCTAGCCCATTAACGCCAAATGATATTCACAAC-3' |
| Rgfp | construct pSK*oriTcat*-pBad-*divIVA-gfp*  and pSK*oriTcat-ptsM-gfp* | 5'-GGTACCTTATTTGTATAGTTCATCCATGCCATGTG-3' |
| Fgfp | construct pSK*oriTcat*-*ptsM-gfp* | 5'-AGATCTAGTAAAGGAGAAGAACTTTTCACTGGAGT-3' |
| RpBad | construct pSK*oriTcat*-pBad-*divIVA-gfp*  and pSK*oriTcat*-pBad-*divIVA-sucA* | 5'-GCTAGCCATTTTTTATAACCTCCTTAGAGCTCGAAT-3' |
| FpBad | construct pSK*oriTcat*-pBad-*divIVA-gfp*  and pSK*oriTcat*-pBad-*divIVA-sucA* | 5'-ATTATGACAACTTGACGGCTACATCA-3' |
| RsucA | construct pSK*oriTcat*-pBad-*divIVA-sucA* | 5'-GGTACCGCTGCAGGCCGCGATCAG-3' |
| FsucA | construct pSK*oriTcat*-pBad-*divIVA-sucA* | 5'-AAGCTTGCAAAGCAAGAACAAGCCCCAGA-3' |
| RptsM | construct pSK*oriTcat*-*ptsM-gfp* | 5'-AGATCTTTTTCCTGTCAGGACCTGGCTT-3' |
| FptsM | construct pSK*oriTcat*-*ptsM-gfp* | 5'-AAGCTTATGATCGGACTCGTGCTTGTT-3' |
| iGal4AD | verify Y2H clones | 5’-CTATTCGATGATGAAGATACCCCACC-3’ |
| Gal4term | verify Y2H clones | 5’- GAAGTGAACTTGCGGGGTTTTTCAG-3’ |
